# Bone Marrow Mesenchymal Stem Cell Hydrogel‐Mediated Fibroblast Reprogramming Restores Intestinal Function in Adhesive Small Bowel Obstruction

**DOI:** 10.1002/advs.202513781

**Published:** 2025-10-27

**Authors:** Lihong Zheng, Junrong Zhang, Zhengyuan Huang, Zhenliang Lin, Jin Zhang, Da Zhang, Ping Hou, Xianqiang Chen

**Affiliations:** ^1^ Department of General Surgery (Emergency Surgery) Fujian Medical University Union Hospital Fujian China; ^2^ Department of General Surgery Fujian Medical University Union Hospital Fujian China; ^3^ Institute of Immunotherapy Fujian Medical University Fujian China; ^4^ The United Innovation of Mengchao Hepatobiliary Technology Key Laboratory of Fujian Province Mengchao Hepatobiliary Hospital of Fujian Medical University Fujian China

**Keywords:** adhesive small bowel obstruction, bone marrow mesenchymal stem cells, fibroblast remodeling, inflammatory response, intestinal repair, TGF‐β/Smad3 signaling pathway

## Abstract

Adhesive small bowel obstruction (ASBO) is a surgical complication characterized by intestinal stenosis due to ectopic fibroblast activation following serosal injury. This study demonstrates that bone marrow mesenchymal stem cells encapsulated in a silk hydrogel (BMSC@Gel) not only prevent tissue adhesion but also restores physiological functions in both mouse tissues and human‐derived organoids. BMSCs exert dual regulatory effects on the obstructed intestinal microenvironment. First, they preferentially differentiate into proliferating fibroblasts (FBs) expressing *Top2a*, *Stmn1*, and *Spp1* rather than inflammatory FBs marked by *Adamdec1, Mmp3, and Igfbp3*. Second, BMSC‐derived exosomes suppress the inflammatory microenvironment, thereby maintaining intestinal homeostasis. Furthermore, BMSCs modulate fibroblast phenotypes and intracellular interactions and inhibit the TGF‐β1/Smad3 signaling pathway during fibrosis development, thereby reversing the onset of ASBO. Collectively, these findings highlight BMSC@Gel as a promising therapeutic strategy for the prevention of ASBO in clinical practice.

## Graphical abstract

Conceptual diagram and schematic illustration of microenvironmental changes in individuals with ASBO before and after treatment. BMSC@Gel therapy transforms the ASBO microenvironment from an inflammatory, fibrotic state with aberrant matrix deposition and barrier dysfunction to a regenerative state with reduced inflammation and improved structural integrity, restoring intestinal homeostasis.

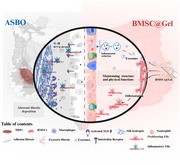

## 1 Introduction

Adhesive small bowel obstruction (ASBO) is a common surgical emergency associated with significant morbidity and mortality, accounting for 20% of all surgical emergencies.^[^ ^1^, ^2^ ^]^ ASBO recurrence imposes severe socioeconomic burdens, with overall national costs for the treatment of patients with ASBO ranging between $3.468 million and $1.77 billion.^[^ ^3^, ^4^ ^]^ Serosal injury derived from intraoperative manipulations, including separation, dissection, ligation, and hemostasis, can activate the inflammatory response and cause fibrotic connections to develop between adjacent bowel tissues.^[^ ^5^, ^6^, ^7^ ^]^ Consequently, this process leads to extracellular matrix (ECM) remodeling (overwhelming cytokine and chemokine production and fibrin deposition) and the aberrant activation of profibrotic fibroblasts, thereby resulting in luminal stenosis and bowel obstruction.^[^ ^8^, ^9^ ^]^ Increased extraluminal mechanical stress and mucosal shrinkage further compromise intestinal physiology, such as mucosal integrity, enteroendocrine system activity, cellular proliferation, and mucin secretion.^[^ ^10^, ^11^ ^]^ The incomplete repair of serosal injury remains a persistent challenge for surgeons, as this injury represents a critical component in the pathophysiology of ASBO.^[^ ^12^ ^]^

The mainstream preventive strategies for ASBO primarily include minimally invasive surgical approaches (e.g., laparoscopic techniques), meticulous operative manipulation (e.g., gentle tissue handling), and antiadhesion barrier applications (e.g., biocompatible materials).^[^ ^13^, ^14^ ^]^ Unsatisfactory effects and severe adverse effects, including restricted manipulation and foreign body reactions, hinder the widespread use of these methods.^[^ ^15^ ^]^ Mesenchymal stem cells (MSCs) are known to perform multiple functions, such as self‐differentiation, self‐renewal, extracellular vesicle production and cytokine alleviation, in the treatment of intestinal injury.^[^ ^16^, ^17^ ^]^ In a dextran sulfate sodium (DSS) ‐induced colitis model, MSCs administered by intravenous injection migrated to the colonic lamina propria and activated myofibroblasts to repair submucosal damage.^[^ ^18^ ^]^ Bone marrow‐derived MSCs (BMSCs), another type of MSC, can treat inflammatory bowel disease (IBD) via immune remodeling and anti‐inflammatory cytokine secretion.^[^ ^16^ ^]^ For local chronic release and better penetration at the injured site, we developed an innovative silk fibroin hydrogel‐mediated delivery system (BMSC@Gel). Given their ultrasonic‐induced spatial variability and superior biocompatibility, these hydrogels provide an optimal 3D cellular niche and excellent tissue integration, thereby effectively restoring both the intestinal barrier and physiological homeostasis.^[^ ^19^ ^]^

Additionally, single‐cell RNA sequencing (scRNA‐seq) is a cutting‐edge technology that allows an in‐depth analysis of the complex cellular composition and heterogeneity within the intestinal microenvironment.^[^ ^20^ ^]^ This technology enables the comprehensive profiling of transcriptional characteristics across various cell types, including intestinal epithelial cells, immune cells, and fibroblasts, providing insights into the dynamic changes and functional states of cell subpopulations in inflammatory bowel disease (IBD) and peritoneal adhesions.^[^ ^21^, ^22^ ^]^ In particular, scRNA‐seq facilitates the identification of primary pathogenic cell subsets, revealing the molecular mechanisms during disease progression.^[^ ^23^ ^]^ Therefore, we utilized scRNA‐seq to characterize the alterations in fibroblast subpopulations following BMSC@Gel treatment and reveal the main pathways through which BMSCs mediate tissue repair.

Herein, we introduce BMSC@Gel designed to reverse fibrin‐mediated luminal stenosis and the inflammatory microenvironment related to the progression of ASBO. BMSC@Gel can modulate fibroblast phenotypes^[^ ^24^, ^25^, ^26^ ^]^ with anti‐inflammatory properties (transition from inflammatory fibroblasts to proliferating fibroblasts), thereby reducing adhesion formation and promoting intestinal repair. Furthermore, we confirmed that BMSCs exert their therapeutic effects by inhibiting the TGF‐β/Smad3 signaling pathway.^[^ ^27^, ^28^, ^29^ ^]^ BMSC@Gel can effectively reverse ASBO formation, laying a solid foundation for the prevention of ASBO in the clinic.

## 2 Results

### 2.1 Characterization of BMSCs and In Vitro Preparation of BMSC@Gel

**Figure 1:**
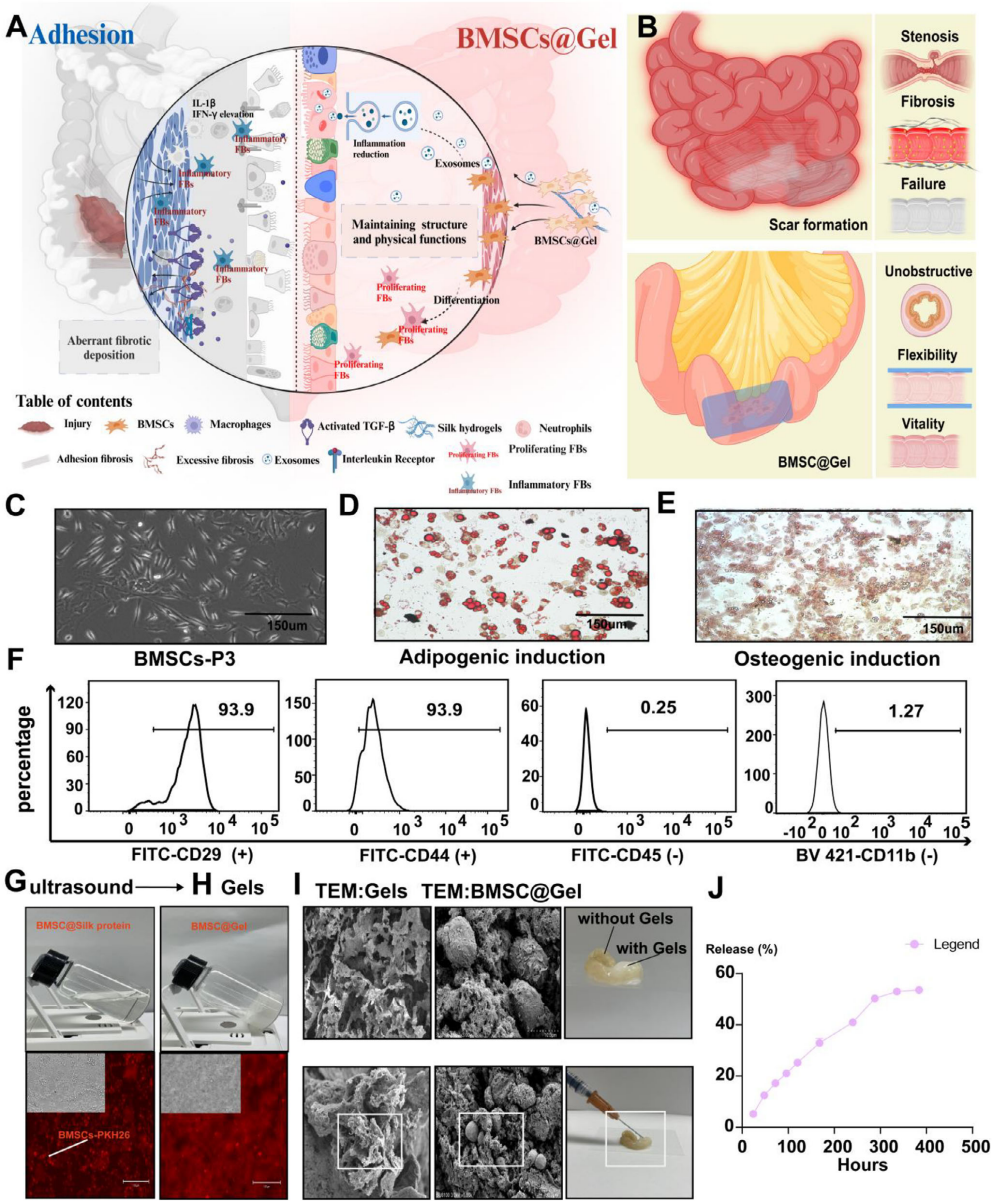
Characterization of the BMSCs and preparation of the BMSC‐loaded hydrogel system with in vitro validation. A,B) Schematic diagram of intestinal injury repair before and after BMSC@Gel treatment. C) Representative phase‐contrast micrographs of cultured BMSCs displaying the characteristic spindle‐shaped morphology (scale bar: 150 µm). D) The adipogenic differentiation capacity was validated by performing Oil Red O staining of intracellular lipid accumulation (scale bar: 150 µm). E) The osteogenic potential confirmed by Alizarin Red S staining of mineralized matrix deposits (scale bar: 150 µm). F) Flow cytometry‐based immunophenotyping showing high expression of the mesenchymal markers CD29 (93.9%) and CD44 (93.9%), with minimal expression of the hematopoietic markers CD45 (0.25%) and CD11b (1.27%). G) Transparent, flowing silk protein and its mixture with PKH‐26‐labeled BMSCs. H) Ultrasound‐treated hydrogels mixed with PKH‐26‐labeled BMSCs show uniform distributions. I) Transmission electron micrographs revealing a preserved nanoporous architecture following BMSC incorporation. J) In vitro kinetics of BMSCs release from hydrogels. The cumulative release reached a plateau (>50%) at 288 h (mean ± SEM, *n* = 3).

Recently, the therapeutic potential of BMSCs in intestinal inflammation has been widely studied, particularly in mouse models and clinical trials of patients with Crohn's disease and ulcerative colitis.^[^ ^30^, ^31^ ^]^ In our study, we extracted BMSCs from mouse femurs to alleviate serosal injury. **Figure** **1**A,B schematically illustrate the development of the ASBO model and subsequent BMSC@Gel therapeutic intervention. In the osteogenic assay, the BMSCs exhibited a typical spindle shape with the formation of calcium nodules; moreover, differentiation into cells containing lipid droplets was observed after stimulation (Figure 1C–E). High levels of CD29 (93.9%) and CD44 (93.9%), which are typical biomarkers for BMSCs, were detected via flow cytometry; conversely, CD45 (0.25%) and CD11b (1.27%) were barely detected on these BMSCs (Figure 1F). In clinical trials, the deterioration of intestinal serosal injury was time‐dependent;^[^ ^32^ ^]^ thus, the slow release of BMSCs into the microenvironment might maximize the desired outcome of adhesion treatment. The PKH‐26‐labeled BMSCs were then integrated with silk protein to form BMSC@Gel, an innovative nanoscale delivery system designed for the sustained and efficient release of BMSCs at injured tissue sites.

A uniform distribution and structural stability were the outstanding points for the clinical translation of the BMSC@Gel for ASBO treatment (Figure 1G–I). The analysis of the release kinetics revealed that BMSCs exhibited time‐dependent release from the hydrogel matrix, reaching a plateau release rate of more than 50% after 12 days (Figure 1J). Our rheological analysis demonstrates that both silk fibroin hydrogels and BMSC@Gel exhibit optimal viscoelastic moduli of ≈1 kPa (Figure S1A–F, Supporting Information), and through CY5‐NHS labeling and in vivo tracking, we further demonstrated controlled degradation kinetics over 14 days (Figure S1G,H, Supporting Information).

### 2.2 BMSC@Gel Prevents ASBO by Reversing Serosal Injury and Prolonging the Survival Time

**Figure 2:**
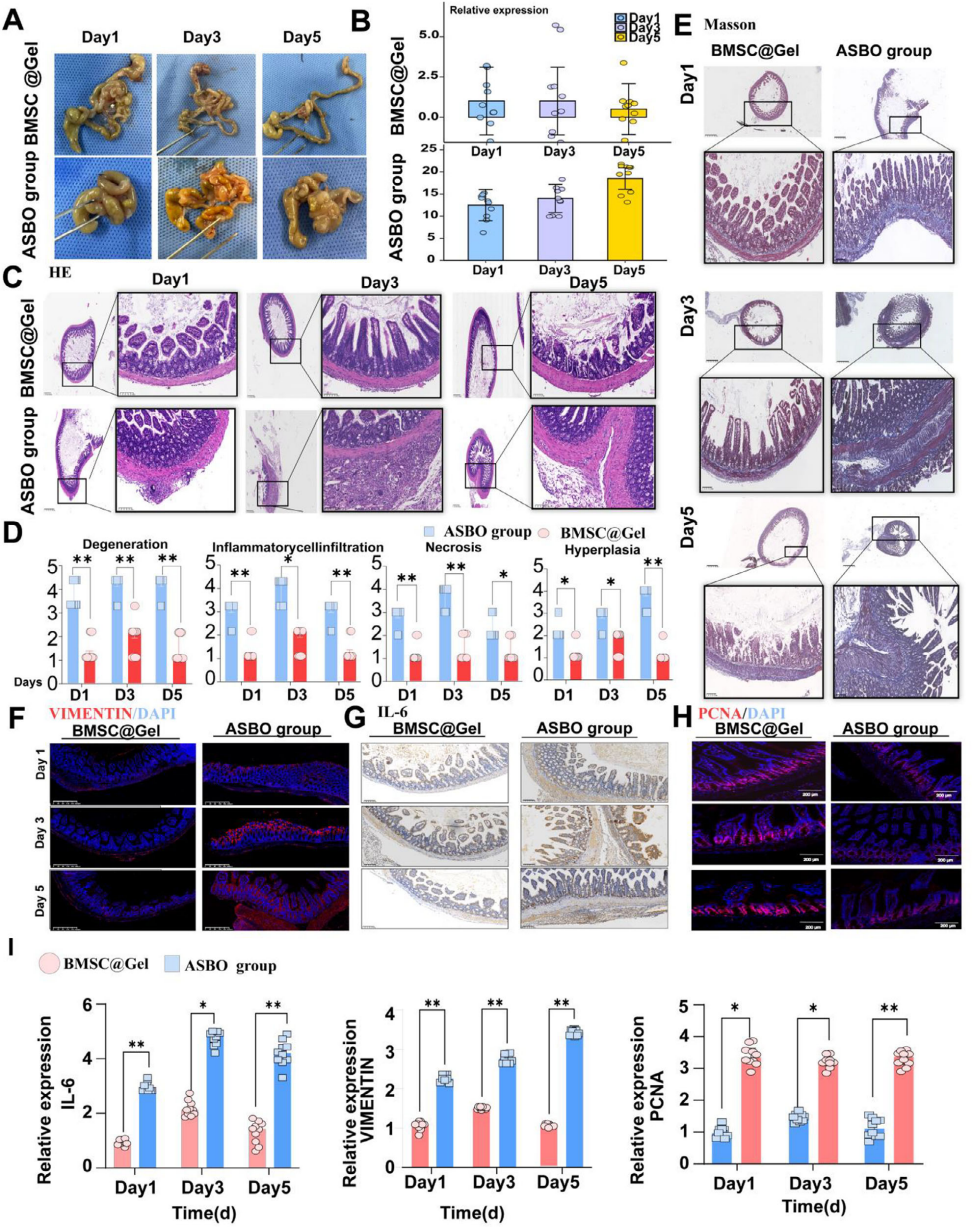
BMSC@Gel attenuated postoperative intestinal obstruction through enhanced tissue repair and modulation of the inflammatory response and intestinal homeostasis. A) Macroscopic evaluation of adhesion and obstruction on Days 1, 3, and 5. The BMSC@Gel intervention preserved the normal intestinal anatomy, whereas the ASBO group (silk‐only) developed progressive adhesion bands with severe intestinal entanglement and edema. B) The quantitative analysis based on the Nair scoring criteria showing significantly reduced adhesion severity in the BMSC@Gel group (grade 0–1.0) compared with the ASBO group (grade 2–4) (*n* = 10 mice per group). All adhesion scores were normalized to the Day 1 BMSC@Gel group mean (set as 1.0) for standardized comparisons. (^****^ *p*<0.0001 for all BMSC@Gel vs ASBO comparisons across all time points). C) Representative images of hematoxylin and eosin staining showing distinct histological outcomes between the groups. BMSC@Gel treatment preserved serosal integrity, physiological submucosal muscle layer thickness and mucosal glandular architecture, in contrast to the progressive mucosal thickening, severe edema, villous atrophy and marked inflammatory infiltration observed in the ASBO group. D) The histological assessment revealed an improved tissue structure and reduced inflammation and fibrosis in the BMSC@Gel group compared with the ASBO group (^*^ *p*<0.05 and ^**^ *p*<0.01). E) Masson's staining revealed a normal collagen architecture in the BMSC@Gel group, whereas the ASBO group presented extensive collagen deposition from the serosa to the subserosa, causing luminal stenosis (scale bar: 100 µm). F) Representative images of immunofluorescence staining for vimentin expression (red, with DAPI counterstaining in blue) on Days 1, 3, and 5, showing attenuated fibroblast activation in the BMSC@Gel group compared with the ASBO group (scale bar = 200 µm). G) Serial immunohistochemical staining demonstrated sustained IL‐6 suppression in the BMSC@Gel‐treated group compared with the ASBO group (scale bar = 100 µm). H) PCNA immunofluorescence staining revealed enhanced tissue regeneration in the BMSC@Gel group, as evidenced by an increase in the number of PCNA‐positive cells at all time points (scale bar = 200 µm). I) Quantitative analysis of vimentin, IL‐6 and PCNA expression levels (^*^ *p* < 0.05 and ^**^ *p* < 0.01, *n* = 10 biologically independent samples per group).

BMSC@Gel maintained >90% BMSCs viability across all time points (24–96 h, Figure S2A–G, Supporting Information), with low ROS production and a high mitochondrial membrane potential (Figure S3A–N, Supporting Information), indicating their general biosafety (Figure S4A–H, Supporting Information). In terms of treatment efficacy, fewer adhesive bands, lower Nair scores (Scores of 0–1, **Figure** **2**A,B), and prolonged survival (Figure S5A, Supporting Information) were observed in the BMSC@Gel group on consecutive days (Days 1–5). In contrast, the ASBO group showed progressive deterioration with increasing Nair scores (scores of 3–4) and high mortality rates (≈75–80% of the mice died on Day 7).

From a microscopic perspective, the integrity of the serosa, the thickness of the submucosal muscle layer, and the glandular structure in the mucosal layer remained stable after BMSC@Gel treatment (Figure 2C). H&E staining and pathological assessments showed that the BMSC@Gel treatment significantly inhibited the degree of tissue injury. Compared with those in the ASBO group, tissue degeneration (ASBO vs BMSC@Gel group, a score of 3–4 vs a score of 1–2), inflammatory cell infiltration (a score of 3–4 vs a score of 1–2), necrosis (a score of 2–4 vs a score of 1), and hyperplasia (a score of 2–4 vs a score of 1–2) were significantly reversed in the BMSC@Gel group (Figure 2D). Severe fibrosis and stiffness were typically observed in the ASBO group, along with the time‐dependent deposition of collagen fibers from the serosa to the mucosa, which was consequently characterized as muscular layer thickening and luminal narrowing. BMSC@Gel treatment dramatically inhibited this progression in a time‐dependent manner (Figure 2E). In addition, VIMENTIN (BMSC@Gel group versus ASBO group, a score of 1–1.39 versus a score of 2.13–3.22), IL‐6 (BMSC@Gel group vs ASBO group, a score of 1–2.30 vs a score of 3.14–5.05) and PCNA (BMSC@Gel group vs ASBO group, a score of 3.19–3.42 vs a score of 1–1.43), were differentially expressed between the groups in a time‐dependent manner (Figure 2F–I). Compared with conventional therapy (BMSC monotherapy and prednisolone) and negative control therapy (PBS, hydrogels, and sham), BMSC@Gel also displayed superior therapeutic efficacy (Figure S5B–K, Supporting Information).

### 2.3 BMSC@Gel Migrates from the Serosa to the Mucosa to Restore Bowel Physiology

**Figure 3:**
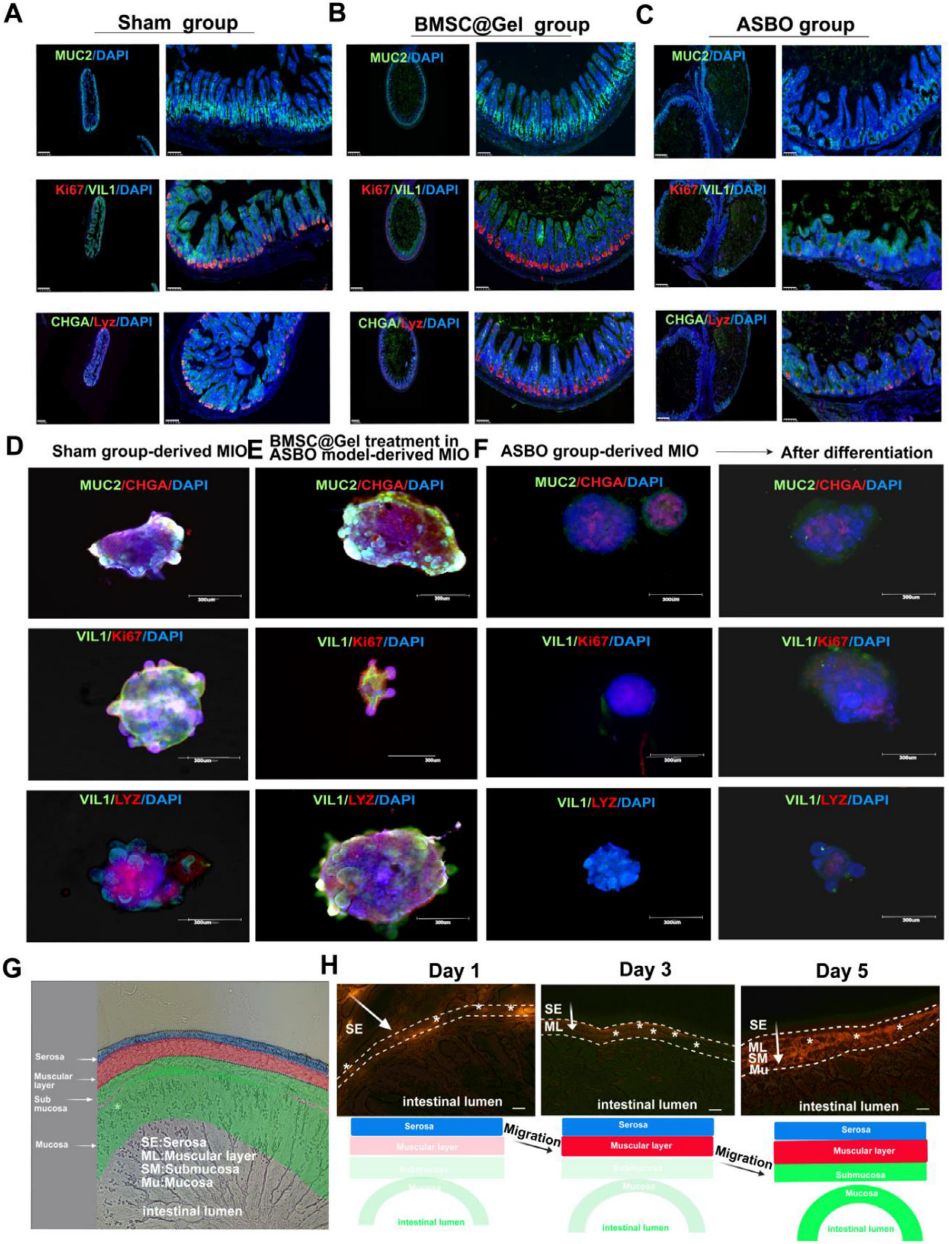
BMSCs migration and intestinal function maintenance. A) MUC2 (mucin, green), Ki67 (proliferation, red), CHGA (enteroendocrine cells, green), VIL1 (enterocyte marker), LYZ (antimicrobial defense), and DAPI (nuclei, blue) expression in intestinal tissues from the sham group of mice (scale bar: 100 µm). B) Expression patterns in BMSC@Gel‐treated tissues were comparable to those in the sham group. C) Significantly decreased marker expression in ASBO mouse intestinal tissues. D) The immunofluorescence analysis of mouse intestinal organoids (MIOs) derived from the sham group and cultured for two weeks revealed expression patterns consistent with those of the sham group tissue. E) The MIO expression levels in the BMSC@Gel treatment group were comparable to those in the organoids from the sham group at the same time points. F) Organoids from the ASBO group showed persistently lower marker expression even after the extended differentiation period (scale bar: 300 µm). G) Schematic illustration of intestinal cross‐sections. H) Migration trajectory of PKH‐26‐labeled BMSCs from the serosa to the submucosa (scale bar: 100 µm).

In contrast to the conventional understanding, our findings first defined adhesive small bowel obstruction (ASBO) not only as a mechanical blockage but also as harmful to bowel physiology via the disruption of the mucosal layer, including the mucosal integrity markers (CDX2 and CDH1), enteroendocrine system (CHGA), cell proliferation (Ki67) and mucin secretion (MUC2) in both the human intestine and parallel organoids (Figure S6A–D, Supporting Information). Similar results were obtained in our mouse models. Compared with that in the sham group, the ablation of the intestinal serosa (establishment of the ASBO group) simultaneously caused mechanical injury and physiological malfunction, especially changes in Ki67, MUC2, CHGA, LYZ, and VIL1 levels. BMSC@Gel reversed these aberrant alterations in the mucosa in a time‐dependent manner (**Figure** **3**A–C). In our mouse organoid model, stem cell differentiation and the fundamental structure of the intestinal mucosa were obviously inhibited in the primary and subsequent ASBO organoids. In parallel, the BMSC@Gel was further shown to repair these disorders and preserve mucosal function (Figure 3D–F). Fluorescence intensity in intestinal organoids derived from ASBO patient tissue was significantly lower than that observed in organoids derived from normal control tissue (Figure S7A,B, Supporting Information).

How do BMSCs dynamically prevent the onset and progression of ASBO? The longitudinal migration analysis revealed that PKH‐26‐labeled BMSCs displayed a classical spatiotemporal distribution: initially accumulating predominantly in the serosal layer at 24 h after transplantation (Day 1), progressively infiltrating the subserosal layer by 72 h (Day 3), and ultimately establishing a stable localization in the mucosal layer by 120 h (Day 5), with a concomitant decreased in the fluorescence intensity in the mucosal layer. This sequential migration pattern from the serosa to the mucosa, verified the capacity of the cells for continuous and transmural repair of the injured intestinal wall (Figure 3G,H).

### 2.4 BMSC‐Derived Exosomes Enhance Intestinal Epithelial Cell Proliferation and Injury Healing

**Figure 4:**
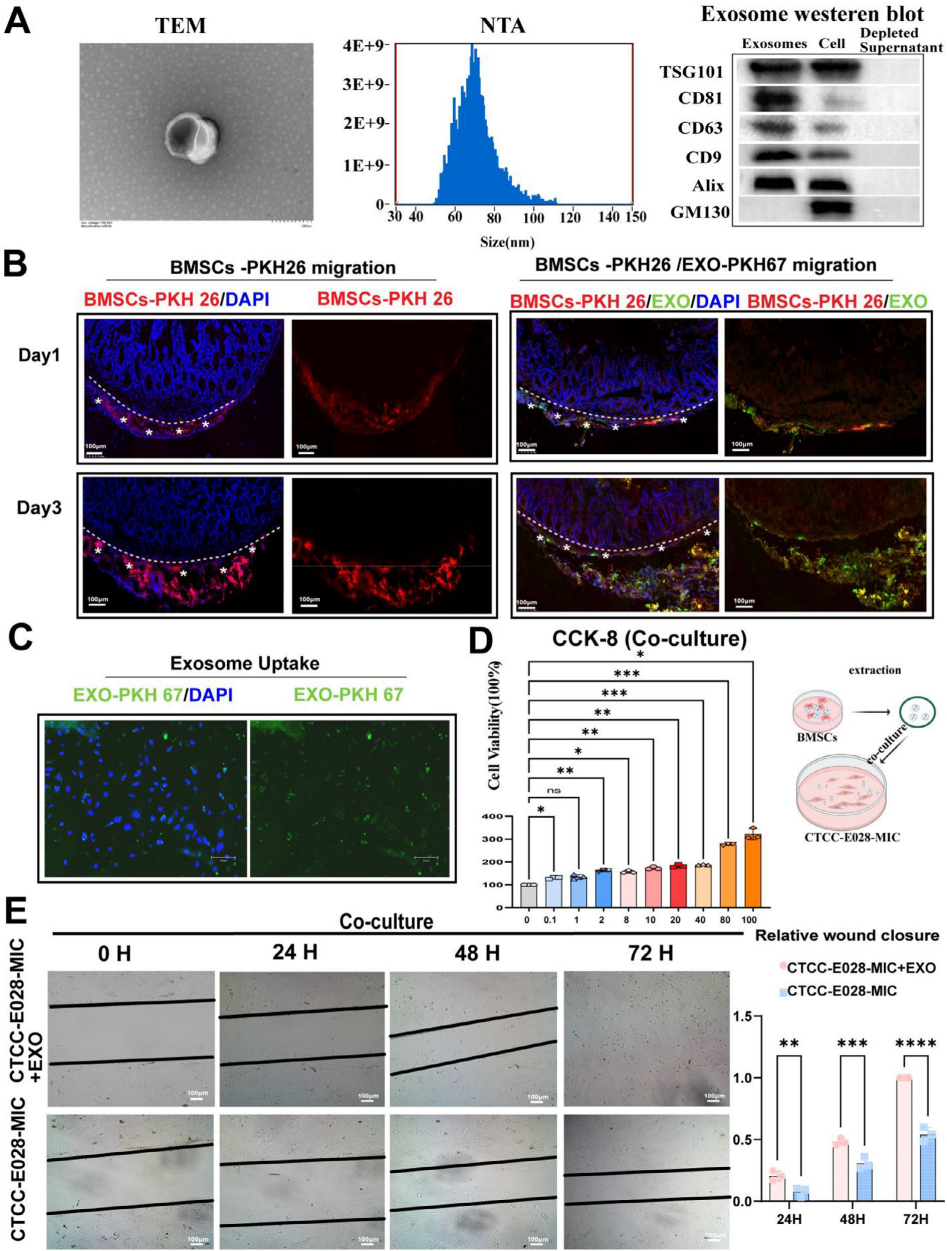
BMSC‐derived exosomes promote intestinal epithelial regeneration: characterization and functional analysis. A) exosome isolation was confirmed by Western blot detection of specific markers (TSG101, CD81, CD63, CD9, Alix), absence of cellular contamination (GM130‐negative), characteristic morphology by TEM, and size range of 50–110 nm by NTA. B) In vivo tracking images showing the distribution of BMSCs (PKH‐26, red) and exos (PKH‐67, green) in mouse intestines on Days 1 and 3 after application, with evidence of colocalization and inward migration (scale bar = 100 µm). C) Representative images showing the absorption of exos (PKH‐67, green) by intestinal epithelial cells in the coculture system. Scale bar = 150 µm. D) Analysis of the proliferation of intestinal epithelial cells treated with different concentrations of BMSC‐exos (0–100 µg mL^−1^) for 24 h (*n* = 3, ^*^ *p*<0.05 and ^**^ *p*<0.01, ^***^ *p*<0.001). E) Wound healing assay of intestinal epithelial cells cocultured with BMSC‐exos (40 µg mL^−1^). Microscopy images showing wound closure at 0, 24, 48, and 72 h postinjury. Compared with the exosome‐free control group, the scratch wound of the BMSC‐exo treatment (40 µg mL^−1^) group had closed at 72 h. The scale bar indicates 100 µm.

According to previous reports, BMSCs can secrete exosomes^[^ ^33^, ^34^ ^]^ to relieve the inflammatory response and enhance intestinal epithelial function repair by migrating to the injured mucosa. In our study, BMSC‐derived exosomes were extracted and thoroughly characterized. Nanoparticle tracking analysis (NTA) revealed that the exosome diameter ranged from 50 to 110 nm, consistent with the typical exosome size distribution. Western blot analysis confirmed the expression of characteristic exosomal markers, including TSG101, CD81, CD63, CD9, and Alix, while demonstrating the absence of the cellular contaminant marker GM130, thereby validating successful exosome isolation and purity (**Figure** **4**A). The distribution of PKH‐26‐labeled BMSCs and PKH‐67‐labeled exosomes colocalized in the same layer of the small intestine in a time‐dependent manner. A similar migration pattern from the serosa (Day 1) to the mucosa (Day 3) was also confirmed for these BMSCs and was specifically accompanied by the secretion of exosomes (Figure 4B). The biological alterations occurring after cells took up BMSC‐derived exosomes were analyzed.^[^ ^35^ ^]^ In our study, CTCC‐E028‐MIC cells showed enhanced proliferation in a dose‐dependent manner (0–100 µg mL^−1^, 24 h; *n* = 3) (Figure 4C–E).

### 2.5 BMSC‐Derived Exosomes Relieve the Intestinal Inflammatory Microenvironment

**Figure 5:**
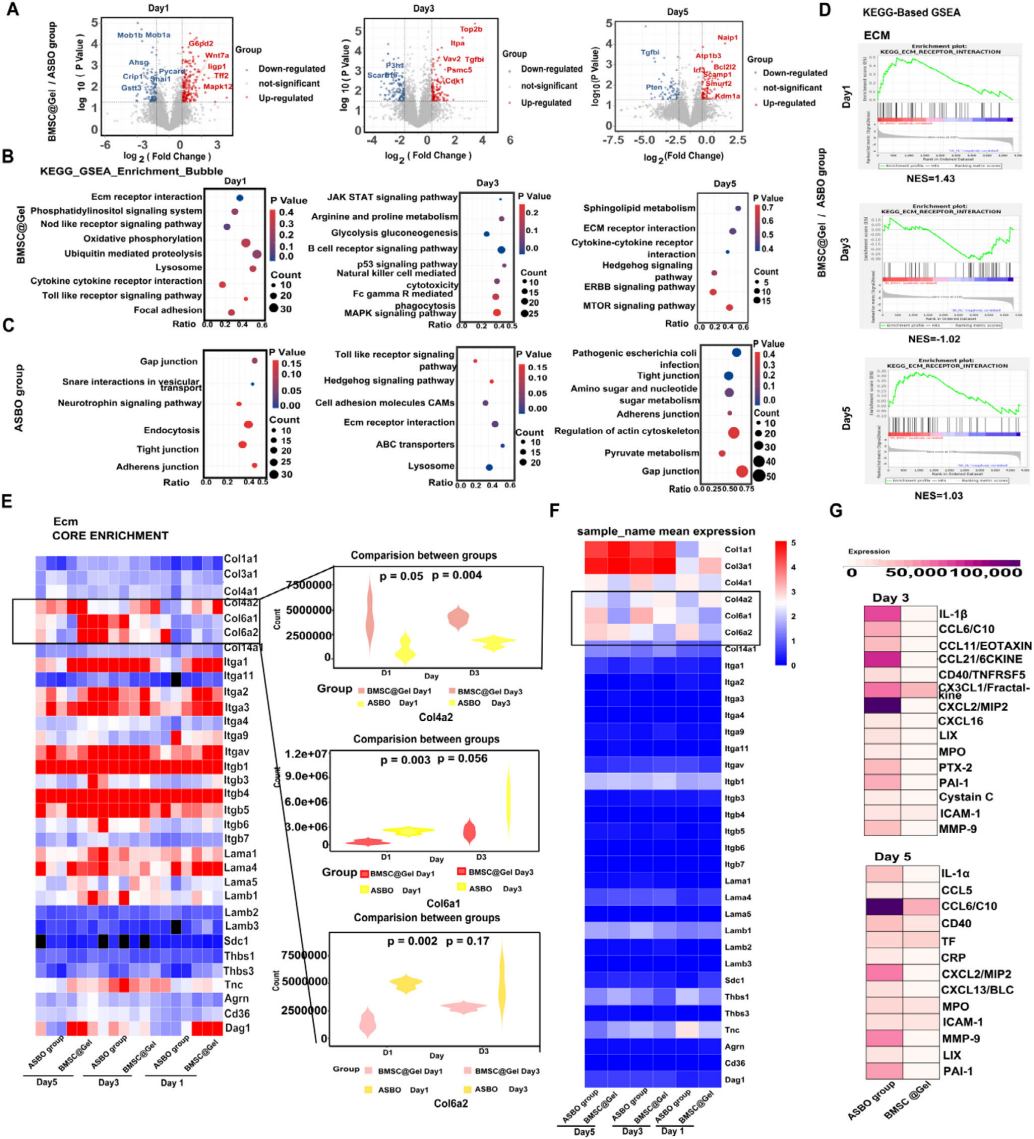
BMSC@Gel‐mediated modulation of the intestinal inflammatory microenvironment via paracrine signaling. A) Volcano plot showing differentially expressed proteins between the BMSC@Gel and ASBO groups (*n* = 3 per group, log_2_|FC|>1.2, *p*<0.05). Upregulated proteins (red) and downregulated proteins (blue) are highlighted. B–D) Gene set enrichment analysis (GSEA) demonstrating the significant enrichment of intestinal barrier maintenance and tissue repair pathways in the BMSC@Gel group (normalized enrichment score >1.5, FDR<0.05) and proinflammatory pathways in the ASBO group. E) Heatmap analysis of ECM‐related core proteins showing the preferential enrichment of Col4a2 (basement membrane collagen) in the BMSC@Gel group versus Col6a1 and Col6a2 (fibrillar collagens) in the ASBO group. The color scale represents log_2_|FC|. F) Multigene expression heatmap across treatment groups and time points, showing similar trends in alterations in ECM remodeling pathways. G) Cytokine array analysis at Days 3 and 5 posttreatment showing that the expression of inflammatory mediators (IL‐1α/β) and matrix metalloproteinase 9 (MMP9) was significantly lower in the BMSC@Gel group relative to the baseline color shown in the heatmap, indicating early repair. The data are presented as the means ± SEMs. FC, fold change.

We explored how BMSC‐derived exosomes prevented the progression of ASBO. Compared with the ASBO group, the BMSC@Gel group exhibited an altered exosomal proteome, with increased secretion of 306‐, 335‐, and 163 proteins on Days 1, 3, and 5, respectively, after the operation. Conversely, the levels of 215‐, 308‐, and 133 proteins were inhibited at the same time points after BMSC@Gel treatment (**Figure** **5**A).

Consistent with previous findings, severe inflammation was initiated upon acute injury to the intestinal serosa, and the production of IL‐1 and IFN‐γ was confirmed via a GSEA‐GO analysis of these proteins in the ASBO group on Day 1. However, the BMSC@Gel significantly inhibited this process, thereby maintaining the physical and physiological functions of the intestine (Figure S8A,B, Supporting Information). On Day 3, fibroblast activation was stimulated following severe inflammation, whereas BMSC@Gel continuously maintained the fundamental physiology.

GSEA revealed distinct ECM–receptor interaction patterns (Figure 5B–D): enhanced intestinal repair on Day 1 (NES = 1.43, *p* = 0.0), suppressed fibroblast activation on Day 3 (NES = −1.02, *p* = 0.40), and increased ECM interactions on Day 5, possibly due to collagen deposition in repaired regions (NES = 1.03, *p* = 0.4). BMSC@Gel could also simultaneously modulate both the Gap and Tight junction pathways in a coordinated temporal manner (Figure S8C,D, Supporting Information). Specifically, BMSC@Gel increased the concentration of *Col4a2* in exosomes on Days 1 and 3. However, BMSC@Gel suppressed the expression of *Col6a1* and *Col6a2*, which are related to aberrant tissue repair and scar formation ^[^ ^36^ ^]^ (Figure 5E). Similar results were further confirmed via Immunofluorescence (IF) of staining in the related tissues (Figure S9A,B, Supporting Information). Furthermore, the gene expression analysis revealed a consistent trend in the expression of ECM‐related core genes (Figure 5F). Compared to ASBO, proinflammatory and fibrosis‐related factors (IL‐1α/β, CXCL2, MMP9, and PAI‐1) were significantly downregulated in the microenvironment of the BMSC@Gel group on Days 3 and 5 using a cytokine array (Figure 5G). In other words, BMSC@Gel initially suppressed both junction formation and excessive fibrosis and then promoted controlled tissue repair to avoid excessive fibrosis.

### 2.6 BMSC@Gel Transforms Inflammatory Fibroblasts Into Proliferating Fibroblasts During the Progression of ASBO

**Figure 6:**
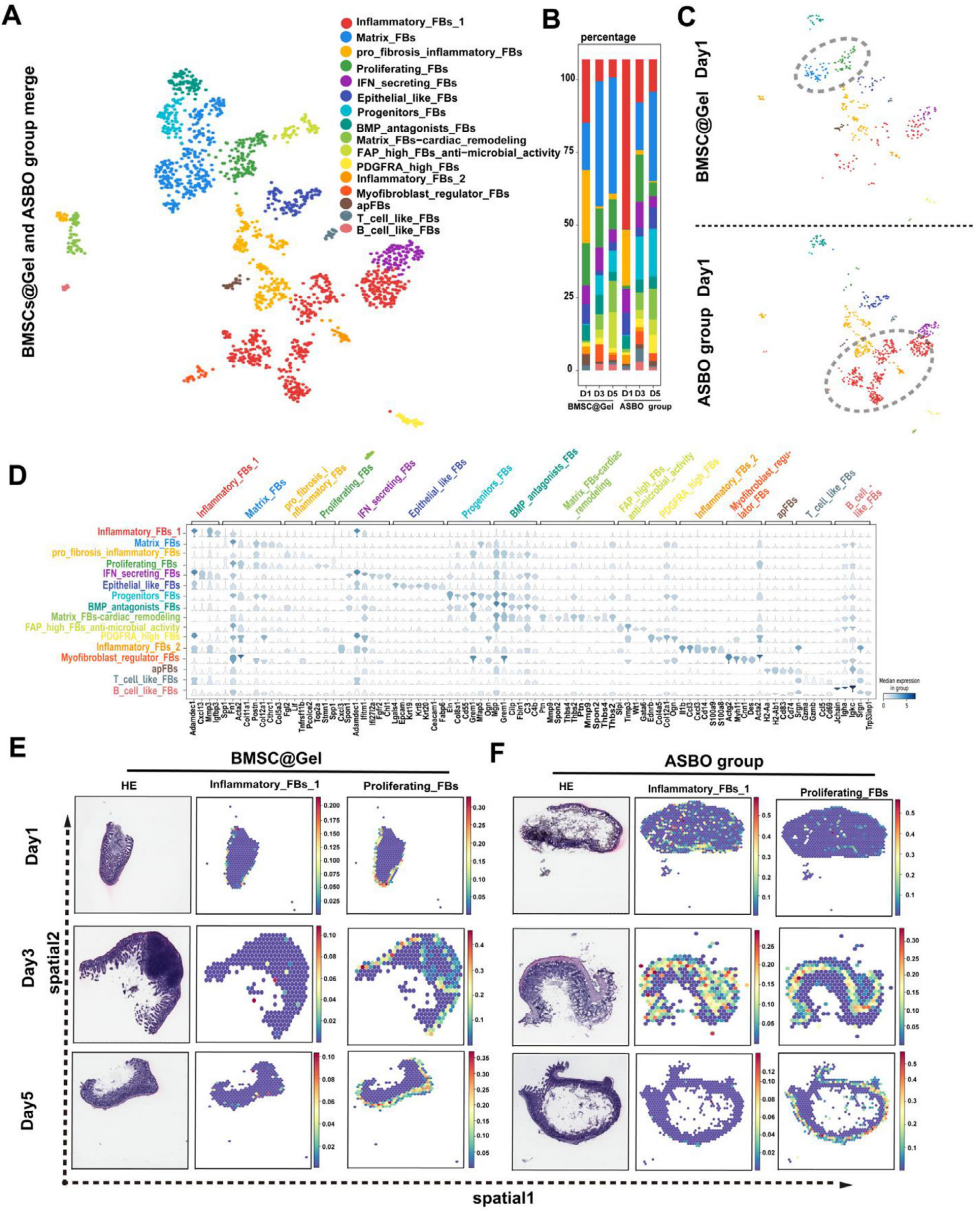
Reprogramming of the fibroblast phenotype by BMSC@Gel during intestinal adhesion: insights from scRNA‐seq. A) UMAP plot showing 16 fibroblast subtype clusters based on gene expression profiles. B) Temporal analysis of the fibroblast subtype composition in the BMSC@Gel group versus the ASBO group (Days 1, 3, and 5). C) UMAP visualization revealing distinct fibroblast (FB) populations on Day 1, with predominant proliferating FBs in the BMSC@Gel group and a substantial elevation of inflammatory FB‐1s in the ASBO group. D) Comparative violin plot analysis showing the expression profiles of key marker genes in distinct fibroblast populations between the BMSC@Gel and ASBO groups. E,F) The spatial transcriptomic analysis revealed distinct fibroblast distributions: the BMSC@Gel group presented decreased inflammatory FB‐1s and increased proliferating FBs in the serosal layer, whereas the ASBO group presented extensive inflammatory FB‐1 infiltration throughout the intestinal wall (Days 1–3). FBs, fibroblasts.

scRNA‐seq was applied to illustrate the complexity and heterogeneity of the murine ASBO model and to determine the impact of the BMSC@Gel on alterations in subclusters of fibroblasts, which are the main component of intestinal serosal fibrosis. The single‐cell sequencing analysis was performed on 1665 fibroblasts, comprising 737 cells from the BMSC@Gel group and 928 cells from the ASBO group. We annotated all fibroblasts (FBs) into 16 subgroups and 24 clusters (*n* = 1665) based on the expression of known marker genes (**Figure** **6**A). The clusters were identified as follows: inflammatory FB‐1s (Clusters 1, 2, 5, 11, and 12; *n* = 104; marked by *Adamdec1*, *Mmp3*, and *Igfbp3*), matrix FBs (Clusters 0 and 8; *n* = 28; marked by *Fn1*, *Col11a1*, and *Postn*), profibrotic inflammatory FBs (Clusters 7, 14, 16, and 21; *n* = 115; marked by *Fgl2*, *Lif*, and *Tnfrsf11b*), proliferating FBs (Cluster 3; *n* = 72; marked by *Top2a*, *Stmn1*, *and Spp1*), IFN‐secreting FBs (Cluster 4; *n* = 48; marked by *Spon1*, *Adamdec1*, and *Ifitm1*), epithelial‐like FBs (Cluster 6; *n* = 14; marked by *Lgals4*, *Epcam*, and *Krt19*), progenitor FBs (Cluster 9; *n* = 31; marked by *Eln*, *Col8a1*, and *Grem1*), BMP antagonist FBs (Cluster 10; *n* = 22; marked by *Mgp*, *Grem1*, and *C3*), matrix FBs for cardiac remodeling (Clusters 13 and 24; *n* = 280; marked by *Ptn*, *Thbs4*, and *Thbs2*), FAP‐high FBs (Cluster 15; *n* = 93; marked by *Slpi*, *Timp3*, and *Wt1*), PDGFRA‐high FBs (Cluster 17; *n* = 23; marked by *Ednrb*, *Col4a5*, and *Ogn*), inflammatory FB‐2s (Cluster 18; *n* = 478; marked by *Il1b*, *Ccl3*, and *Cxcl3*), myofibroblast regulatory FBs (Cluster 19; *n* = 68; marked by *Actg2*, *Myh11*, and *Cnn1*), T‐cell‐like FBs (Cluster 22; *n* = 200; marked by *Srgn*, *Ccl5*, and *Cd69*), and B‐cell‐like FBs (Cluster 23; *n* = 71; marked by *Igha*, *Igkc*, and *Srgn*), along with other cells (*n* = 18). The subgroup compositions varied at different time points (Figure 6B,D). Notably, significant alterations in fibroblast subtype proportions were observed between the ASBO and BMSC@Gel groups, particularly for inflammatory FB‐1s (Clusters 1, 2, 5, 11, and 12) and proliferating FBs (Cluster 3). Unlike inflammatory FB‐1s, which were instantly activated upon injury to the intestinal serosa in the ASBO group, BMSC@Gel treatment distinctly remodeled the subcluster of fibroblasts into proliferating FBs (Figure 6C), with a time‐dependent decrease in inflammatory FB‐1s accompanied by an increase in proliferating FBs (Figure S10A,B, Supporting Information). In accordance with the exosome proteomic results, *Col4a2* was localized to proliferating FBs, whereas *Col6a1* and *Col6a2* were specifically localized to inflammatory FBs (Figure S10C, Supporting Information). The spatial distribution analysis also indicated that inflammatory FB‐1s were less abundant in the serosa in the BMSC@Gel group than in the ASBO group on Day 1, whereas proliferating FBs were more abundant in the serosa. In accordance with the BMSCs migration trajectory, proliferating FBs migrated from the serosa to the subserosa on Day 3. The inflammatory FB‐1s expanded within the intestinal wall within 3 days in the ASBO group; however, this population vanished in each layer of the small intestine in the BMSC@Gel group (Figure 6E,F).

### 2.7 Distinct Roles of Inflammatory Fibroblasts And Proliferating Fibroblasts in the Progression of ASBO

**Figure 7:**
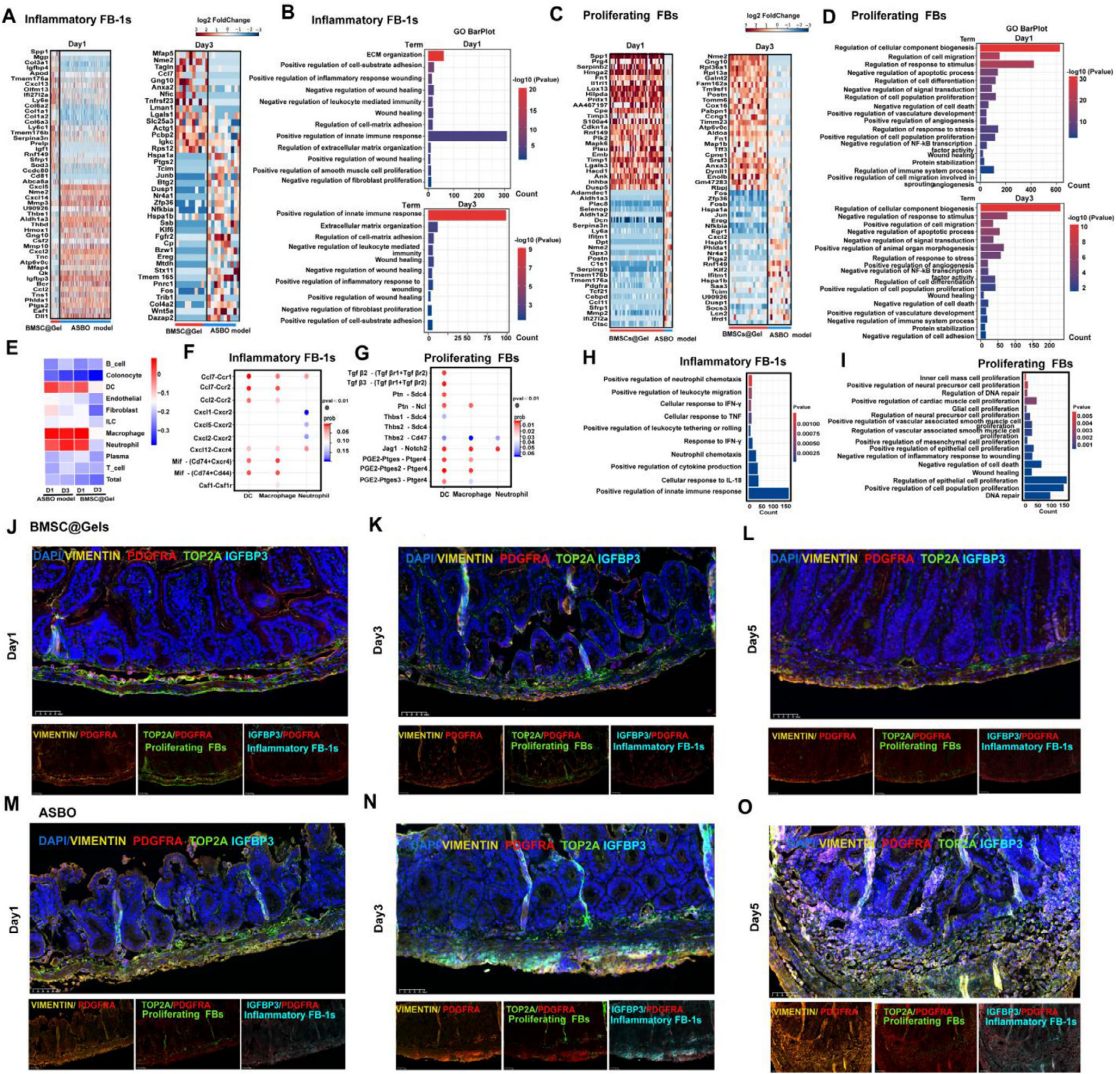
Functional characterization of inflammatory fibroblasts and proliferating fibroblasts through Gene Ontology annotation. A,B) Heatmap showing differential gene expression profiles of inflammatory fibroblast subtype 1 (FB‐1s) between the BMSC@Gel‐treated and ASBO groups. Gene Ontology (GO) enrichment analysis revealed that inflammatory FB‐1s were predominantly associated with the ECM and inflammatory pathways. C,D) Heatmap displaying distinct gene expression patterns of proliferating fibroblasts between the treatment groups. The GO analysis revealed that proliferating FBs were enriched in the tissue repair pathway. E) Interactions between FBs and innate immunocytes. F,G) The cell‒cell interaction analysis indicated distinct signaling patterns: inflammatory FBs predominantly activated proinflammatory pathways (including the CCL, CXCL, MIF, and CSF families), whereas proliferating FBs primarily enhanced the TGF‐β, NOTCH, and prostaglandin family signaling pathways. H,I) The GO analysis showed that inflammatory FBs were enriched in pathways related to the immune response and cytokine regulation, whereas proliferating FBs were enriched in pathways associated with smooth muscle and epithelial cell proliferation, inner cell mass proliferation, and wound inflammation suppression pathways (*p* < 0.01). J–L) Immunofluorescence analysis of fibroblast phenotypes in the BMSC@Gel group on Days 1, 3, and 5 posttreatment. Images show predominant proliferating FBs (*TOP2A+/PDGFRA+*) with minimal inflammatory FBs (*IGFBP3+/PDGFRA+*). DAPI (blue) indicates nuclei. Scale bars = 50 µm. M–O) Immunofluorescence analysis of fibroblast phenotypes in the ASBO group on Days 1, 3, and 5. The images show dominant inflammatory FBs (*IGFBP3+/PDGFRA+*) with a significantly increased overall fluorescence intensity, indicating increased fibroblast activation and recruitment to adhesion sites. Scale bars = 50 µm, FBs, fibroblasts.

GO annotations were determined to further illustrate the different functions of inflammatory FB‐1s and proliferating FBs between the groups, and the results revealed that inflammatory FB‐1s primarily promoted ECM stiffness (*Mmp10*, *Thbs1*, and *Mfap4*) and wound inflammation (*Cxcl5* and *Cxcl14*) in the ASBO group, which was reprogrammed via ECM organization (*Col3a1* and *Col6a2*) and tissue repair regulation (*Igfbp4* and *Cxcl13*) after BMSC@Gel treatment (**Figure** **7**A,B). The proliferating FBs in the BMSC@Gel group expressed repair‐related genes. Conversely, proliferating FBs in the ASBO group presented inflammatory activation (*Serping1*, *C1s1*, and *Ifitm1*), acute stress responses (*Fos, Jun*, and *Egr1*), proinflammatory signaling (*Cxcl2* and *Saa3*), and heat shock responses (*Hspa1a* and *Hspb1*) (Figure 7C,D). The crosstalk analysis further confirmed that BMSC@Gel significantly inhibited the interaction between FBs and innate immunocytes, including macrophages, DCs, and neutrophils (Figure 7E). Moreover, inflammatory FB‐1s exerted proinflammatory effects primarily via proinflammatory chemokine axes (Ccl7–Ccr1/2 and Ccl2–Ccr2) and cytokine signaling complexes (MIF–(Cd74+Cxcr4) and MIF–(Cd74+Cd44)), increasing neutrophil recruitment and inflammation through TNF and IFN‐γ. In contrast, proliferating FBs exerted tissue‐reparative effects by activating the Tgfb2/3–(Tgfbr1+Tgfbr2), Jag1–Notch2, and PGE2–Ptger4 signaling pathways, thereby promoting smooth muscle cell and epithelial cell proliferation while suppressing cell death (Figure 7F–I).

Distinct fibroblast phenotype distributions were observed between the groups. In the BMSC@Gel group, proliferating FBs (TOP2A+/PDGFRA+) predominated, with a minimal presence of inflammatory FBs (IGFBP3+/PDGFRA+), and the overall fluorescence intensity remained relatively low at all time points (Figure 7J–L). In contrast, the ASBO group exhibited a marked increase in inflammatory FBs compared to the BMSC@Gel group (Figure 7M–O). Obviously, different differentiation trajectories of FBs were observed between the ASBO and BMSC@Gel groups.

Regarding the distinct differentiation trajectories, inflammatory FB‐1s (*Adamdec1+*) in the ASBO group progressed toward pathological IFN‐secreting fibroblasts, whereas proliferating fibroblasts (*Spp1+*) differentiated into beneficial matrix fibroblasts after BMSC@Gel treatment (Figure S11A,B, Supporting Information). In terms of the temporal fate redirection, the pseudotime analysis demonstrated that ASBO fibroblasts advanced to late‐stage pathological phenotypes, whereas BMSC@Gel‐treated fibroblasts remained in early, more homeostatic differentiation states (Figure S11C–E, Supporting Information). Regarding pathway‐level reprogramming, the analysis of ECM‒receptor interaction pathways showed distinct enrichment patterns between the treatment groups, indicating fundamental changes in cellular programming (Figure S11F, Supporting Information).

### 2.8 BMSC@Gel Promotes Intestinal Repair Through the Inhibition of the TGF‐β1/Smad3 Signaling Pathway

**Figure 8:**
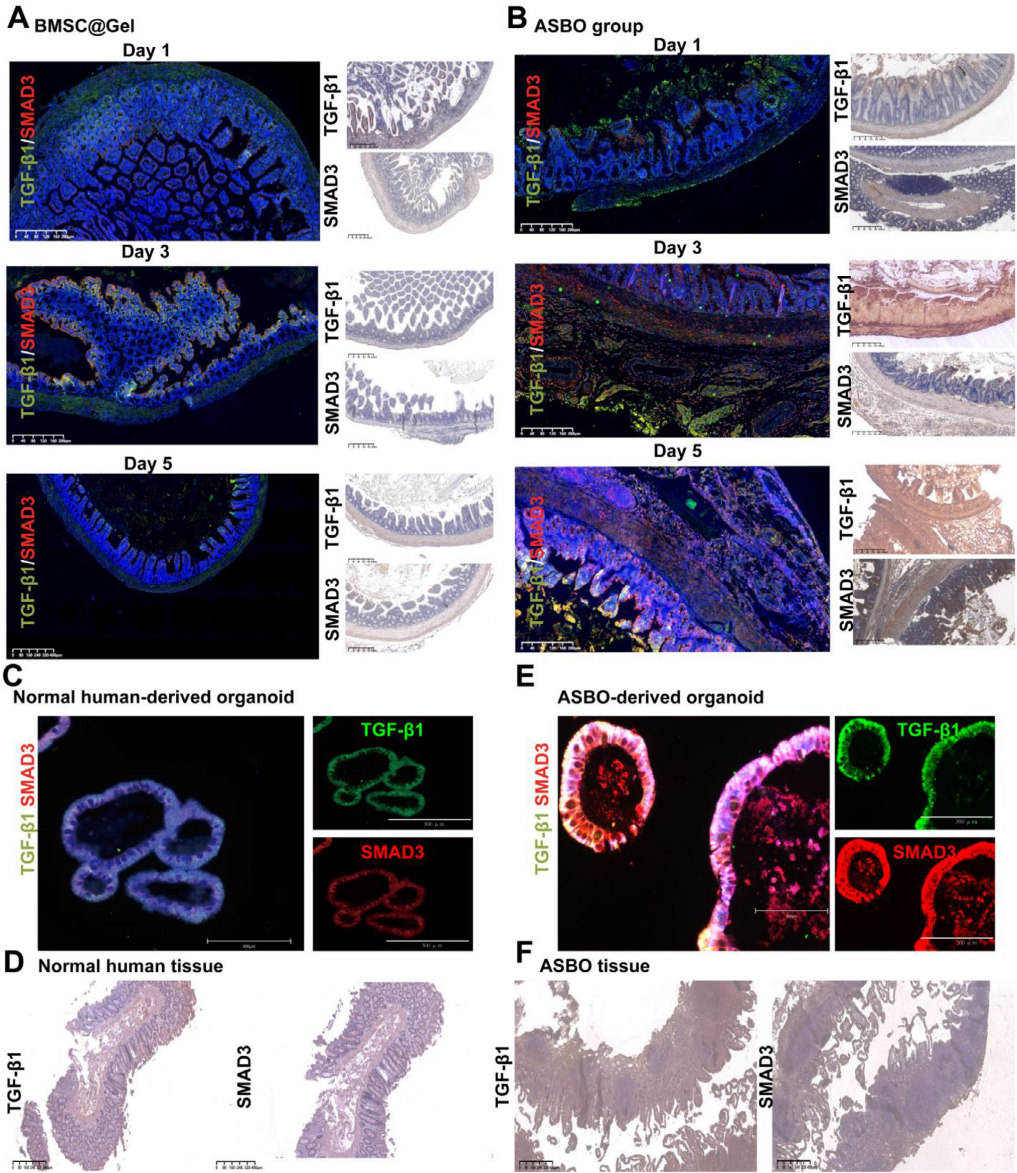
The TGF‐β‒Smad3 signaling axis is critical in ASBO pathogenesis. A) Representative images of immunofluorescence (IF) staining and immunohistochemistry (IHC) showing the attenuated expression of TGF‐β1 and SMAD3 in mouse intestinal tissue following BMSC@Gel treatment. B) Representative images of IF staining and IHC showing increased expression of TGF‐β1 and SMAD3 in the ASBO group compared with the BMSC@Gel‐treated group. C,D) Representative images of IF staining of organoids derived from normal human intestinal tissue showing low expression of TGF‐β1 and SMAD3, with corresponding IHC staining of matched tissue samples confirming minimal pathway activation. E,F) Representative images of immunofluorescence (IF) staining showing significantly increased TGF‐β1 and SMAD3 expression in human ASBO tissue‐derived organoids compared with normal human intestinal tissue‐derived organoids, with the corresponding immunohistochemical (IHC) analysis of tissue samples confirming increased pathway activation.

Previous studies have established that distinct fibroblast populations play dual roles in either preventing or promoting intestinal adhesion formation. The TGF‐β/Smad3 signaling pathway has been widely recognized as a critical mediator of fibrosis.^[^ ^37^, ^38^, ^39^ ^]^ To further investigate the mechanism by which BMSC@Gel reverses fibrotic progression in ASBO, we analyzed the expression of TGF‐β and SMAD3 in both mouse models and human ASBO organoids using IF and IHC staining. Notably, BMSC@Gel consistently attenuated the expression of both SMAD3 and TGF‐β1 during the dynamic progression of ASBO in the mouse model (**Figure** **8**A,B). Compared with paired normal intestinal tissues, human ASBO organoids presented markedly elevated TGF‐β1 and SMAD3 expression (Figure 8E,F), whereas minimal expression was confirmed in normal human intestinal organoids (Figure 8C,D). TGF‐β inhibition (SB‐431542) was also confirmed that BMSCs orchestrate fibroblast phenotype via TGF‐β/Smad3, blockage of this pathway would hinder the differentiation of inflammatory FBs (Figure S12A–C, Supporting Information). CellChat analysis further revealed bidirectional interaction between inflammatory and proliferative FB subpopulations, especially via TGF‐β signaling pathways that were activated in inflammatory FBs (Figure S13A–D, Supporting Information).

## 3 Discussion

Adhesive small bowel obstruction (ASBO) is a complex pathological process initiated by serosal injury that triggers the cascading activation of chemokines and cytokines.^[^ ^40^ ^]^ In our study, this injury promoted the differentiation of fibroblasts into inflammatory clusters characterized by elevated expression of *Adamdec1*, *Mmp3*, and *Igfbp3*. These activated fibroblasts act as negative regulators of wound healing by promoting excessive ECM deposition.^[^ ^41^ ^]^ Critically, the intricate crosstalk between inflammatory cells and fibroblasts plays a pivotal role in adhesion formation,^[^ ^42^ ^]^ leading to abnormal tissue repair characterized by excessive fibrosis, scar formation, and aberrant wound healing.^[^ ^43^ ^]^ Our findings demonstrated that in the ASBO group, the serosal and muscular layers exhibited abnormal thickening, with greater inflammation and a degree of injury that was twice that of the BMSC@Gel group in our study, as determined by H&E staining. Severe widespread fibrosis was also confirmed in the ASBO group, in accordance with previous findings that adhesion formation results from the overwhelming activation of collagen fibers.^[^ ^44^ ^]^ A ≈6‐fold increase in IL‐6 levels was observed in the ASBO group,^[^ ^45^ ^]^ indicating a progressively increasing trend and further substantiating the fibrotic cascade. Similarly, significant accumulation of chemokines, including CCL21, IL‐1β/α, and CCL6, was detected in the pathological microenvironment of the ASBO group by cytokine array.^[^ ^46^ ^]^ Additionally, increased expression of IL‐1, IFN‐γ, and TNF superfamily cytokines was observed according to exosome proteomic analysis.^[^ ^47^, ^48^, ^49^ ^]^ Furthermore, based on the scRNA‐seq analysis, a novel pathogenic fibroblast phenotype (inflammatory FB‐1s) was identified in the injured serosa, with peak expression on Days 1 and 3 postinjury, subsequently terminating in IFN‐secreting fibroblasts. These inflammatory FBs play a key role in adhesion ^[^ ^50^ ^]^ which can lead to obstruction and enhance the inflammatory response, impeding wound healing. Spatial transcriptomics confirmed widespread inflammatory FBs in the ASBO group, which were widely distributed in the serosa and subserosa and peaked on Day 3.^[^ ^51^, ^52^ ^]^

BMSCs exert therapeutic effects on IBD through multiple mechanisms. First, BMSCs possess both potent immunomodulatory functions and participate in intracellular communication to enhance epithelial cell proliferation and repair.^[^ ^53^, ^54^, ^55^ ^]^ Silk fibroin gels are natural protein‐based biopolymers with hierarchical mesoscopic structures containing β‐crystallites and nanofibrils, exhibiting biomimetic extracellular matrix (ECM) properties.^[^ ^56^, ^57^, ^58^ ^]^ Silk fibroin gels possess tunable mechanical characteristics and enable live cell encapsulation, making them suitable for 3D cell culture and tissue engineering. Compared to other biopolymers, silk fibroin gels' unique structural assembly provides superior mechanical performance, excellent biocompatibility, controllable biodegradation, aqueous processability, and minimal inflammatory response.^[^ ^59^, ^60^, ^61^ ^]^ Additionally, silk fibroin materials are cost‐effective and easy to handle, with degradation products that promote stem cell proliferation.^[^ ^62^ ^]^ Owing to these excellent biocompatibility and tunable mechanical properties, Silk fibroin gels function as effective scaffolds for drug delivery and tissue regeneration ^[^ ^63^ ^]^ and can serve as an optimal vehicle for bone marrow mesenchymal stem cells (BMSCs), such as BMSC@Gel constructs, establishing Silk fibroin gels as promising biomedical platforms. The sustained release of BMSCs from the silk fibroin gel can reverse pathological features to recover the intestinal structure, minimize the thickness of each layer, and alleviate severe inflammation. Mechanistically, BMSCs can migrate from the serosa to the mucosa and differentiate into reparative fibroblasts (proliferating FBs in our study, and matrix FBs in the terminal state), confirming their ability to migrate to the site of injury.^[^ ^64^, ^65^ ^]^ Unlike inflammatory clusters, this cluster contributes to structural tissue reconstruction, wound healing promotion, and tissue tension maintenance, thus facilitating repair. In addition, paracrine exosomes derived from BMSCs promote mucosal cell proliferation and wound healing to enhance improvements in both the mucosal and serosal layers. Previous investigations have demonstrated that CD34+ Gp38+ nonmyofibroblastic mesenchymal cells exhibit analogous functions in maintaining intestinal microenvironmental homeostasis and counteracting inflammatory responses postinjury.^[^ ^66^ ^]^ In parallel, attenuated inflammatory cell infiltration characterized by downregulated IL‐6 and chemokine expression, along with the reversal of aberrant fibrotic patterns, was confirmed by H&E staining. Exosomes derived from the BMSC@Gel could enhance gastrointestinal epithelial integrity and structural homeostasis. Depletion of BMSC exosomes would partly attenuate the therapeutic efficacy of BMSC@Gel (Figure S14A–C, Supporting Information). The single‐cell sequencing analysis showed that rather than an increase in inflammatory FBs, a predominant increase in proliferating FBs was observed from Day 1 onward, which progressively contributed to the centripetal tissue repair process. Moreover, BMSCs effectively promote this beneficial transformation through the protein‐mediated suppression of inflammation and modulation of fibroblast phenotypes.

Unlike the conventional concept that ASBO is just defined as blockage of the intestine, strikingly, a more complicated physiological disruption in intestinal functions (proliferation, stemness, endocrine secretion, and reabsorption) was identified in our study. BMSC@Gel treatment could reverse this physiological dysfunction in both mouse and human samples, which has also been reported in skin regeneration.^[^ ^67^ ^]^ Notably, BMSC@Gel effectively restored mucosal barrier integrity in both mouse models and organoid systems, demonstrating their ability to reverse physiological dysfunction and preserve intestinal stem cell populations. Conventional adhesion prevention strategies, including hyaluronic acid‐based barriers and Seprafilm, primarily rely on passive “physical separation” mechanisms. However, the rigid membrane structure of Seprafilm poses significant application challenges through laparoscopic ports during minimally invasive surgical procedures.^[^ ^68^, ^69^ ^]^ Moreover, surgical adhesiolysis and systemic corticosteroid use for inflammation control are other methods for the treatment of ASBO. However, passive physical deterioration and severe side effects hinder the widespread use of these therapies. In contrast, our BMSC@Gel system functions as “bioactive repair,” more than “physical separation,” and represents a novel strategy through its multiple biofunctions. Immune modulation, reprogramming of fibroblast phenotypes, and controlled release of BMSC‐derived exosomes to alleviate inflammation constitute distinctive advantages over conventional strategies. Additionally, prolonged local retention, stable cell viability of BMSCs, and sustained regulation throughout the intestinal repair further illustrate the benefits of using BMSC@Gel as an innovative strategy for the prevention and treatment of ASBO. This multifaceted approach not only improved survival outcomes in mouse models but also exerted superior anti‐inflammatory and pro‐regenerative effects through targeted therapy with minimal systemic side effects.

While this study provides valuable insights into the mechanism and treatment of ASBO, its limitations require formal recognition and should be addressed in future studies. 1) Immunogenicity and safety considerations: although the mouse model presented good biocompatibility of BMSC@Gel, the long‐term immunogenicity of allogeneic bone marrow mesenchymal stem cells remains unknown. Chronic immune responses, the potential for ectopic tissue formation, and the immunosuppressive effects of BMSCs warrant systematic investigation in immunocompetent models prior to clinical translation. 2) Manufacturing scalability and standardization: the clinical translation of BMSC@Gel faces significant manufacturing challenges, including: a) standardization of BMSC isolation and expansion protocols; b) establishment of consistent quality control metrics; c) development of cost‐effective large‐scale production methods; and d) ensuring batch‐to‐batch reproducibility. 3) Regulatory and translational barriers: BMSC‐based therapies face complex regulatory pathways, including investigational new drug applications, extensive preclinical safety studies, and multi‐phase clinical trials. 4) Realistic clinical implementation: our study only represents an early‐stage proof‐of‐concept investigation. Realistic clinical translation would be required. 5) Future developments in oral delivery systems would represent a promising avenue for clinical translation and need to address challenges, including limited bioavailability, gastrointestinal stability, and targeted delivery.

In conclusion, this groundbreaking study provides novel insights into the pathophysiology of ASBO and establishes BMSC@Gel as an innovative therapeutic strategy. BMSC@Gel has remarkable therapeutic efficacy through multiple mechanisms: 1) suppressing inflammatory FBs while promoting the outgrowth of proliferating FBs; 2) inhibiting inflammatory pathways through the downregulation of inflammatory factors such as IL‐1, IFN‐γ, and IL‐6 to maintain the stability of the intestinal structure; and 3) restoring both the physical and chemical barriers of the intestine, as evidenced by the recovery of crucial physiological biomarkers and preservation of intestinal stem cell populations, as validated in both mouse models and organoid systems. This comprehensive investigation not only advances our fundamental understanding of the pathophysiology of ASBO but also establishes BMSC@Gel as a promising therapeutic approach for clinical translation, representing a significant advancement in gastrointestinal medicine.

## 4 Experimental Section

### Isolation and Characterization of Bone Marrow Mesenchymal Stem Cells (BMSCs)

BMSCs were isolated from the femurs of 3–4‐week‐old male C57BL/6 mice (Wushi Laboratory Animal Co., Ltd., Shanghai, China) via differential adhesion and cultured in α ‐MEM(Gibco, USA) supplemented with 10% FBS (Sigma, USA) at 37 °C with 5% CO_2_.^[^ ^70^ ^]^ Cells at passages 3–5 were characterized by morphological assessments using phase‐contrast microscopy (Nikon Eclipse Ti, Japan), flow cytometry analyses of surface markers (CD45, CD44, CD29, and CD11b, shown in Table S2, Supporting Information). Analysis was performed using the BD FACSVerse and the FlowJo software. Differentiation potential was verified by inducing differentiation toward osteogenic (21 days, Alizarin Red S staining, OriCell, China, Cat# MUXMX‐90021) and adipogenic (14 days, Oil Red O staining, OriCell, China, Cat# MUXMX‐90031) lineages.

### Synthesis of the BMSC@Gel

Silk fibroin hydrogels were prepared according to previously published protocols with slight modifications.^[^ ^71^ ^]^ Briefly, silk cocoons (5 g) were cut into pieces, boiled in a 20 mm Na_2_CO_3_ solution for 30 min, and rinsed three times with ddH_2_O to remove sericin proteins. The extracted silk fibroin was air‐dried at 60 °C for 12 h, then dissolved in 9.3 m LiBr salt solution at 60 °C for 4 h, followed by dialysis (MWCO 3.5 kDa) against distilled water for 72 h to remove the LiBr salt. The obtained silk fibroin solution was purified by centrifugation at4 °C and stored at 4 °C until further use. The purified silk fibroin solutions were degummed and sonicated using an ultrasonic probe (Scientz‐IID, Ningbo Scientz Biotechnology, China) at 30% amplitude for 180 s, followed by aging at 37 °C to prepare 2 wt.% silk hydrogels. Before preparing the BMSC@Gel constructs, the BMSCs were first suspended in PBS (Gibco, USA) at 4 °C to maintain cell viability. The pregelled silk fibroin solution (2 wt.%, maintained at 4 °C) was gently mixed with the BMSC suspension using a micropipette with slow aspiration and expulsion (10 cycles) to ensure a homogeneous cell distribution while avoiding mechanical damage. The cell–hydrogel mixture was maintained at 4 °C during preparation to prevent premature gelation. The final cell density was adjusted to 3 × 10^7^ cells mL^−1^. The mixture was then immediately transferred to 1.5 mL Eppendorf tubes and allowed to gel at 37 °C for 10 min. Cell viability after encapsulation was confirmed to be >95% using live/dead staining (calcein‐AM/propidium Iodide, Dojindo Laboratories, Kumamoto, Japan, Cat# C542). The BMSC@Gel constructs were used immediately for implantation. The distribution of PKH26 (Sigma, Cat# PKH26GL)‐labeled BMSCs within the hydrogel was visualized using a fluorescence microscope. For the ultrastructural analysis, the samples were fixed, dehydrated, and subjected to critical‐point drying, followed by gold sputter coating and examination using a JEM1400 transmission electron microscope (JEOL, Japan) at an accelerating voltage of 80 kV.

To track silk fibroin hydrogel biodistribution and accumulation in vivo, silk fibroin proteins were covalently labeled with CY5‐NHS ester using standard bioconjugation protocols (protein concentration: 2 mg mL^−1^, pH 8.5, CY5‐SE:protein molar ratio 10:1, room temperature conjugation for 60 min), followed by purification using SepHadex G‐25 columns, and the labeled hydrogels were quantitatively tracked using IVIS Lumina X5 imaging system (Perkin Elmer) with Living Image software for data acquisition and GraphPad Prism for statistical analysis, *n* = 3/days.

### Rheological Characterization of Silk Fibroin Hydrogels

The rheological properties of silk fibroin hydrogel precursors were characterized using a rotational rheometer (MCR92, Anton Paar, Austria). Hydrogel samples were carefully placed on the sample stage using a spatula to avoid air bubble incorporation. Measurements were performed using a 50‐mm parallel plate geometry with a gap setting of 1 mm. Frequency sweep tests were conducted in the linear viscoelastic region with a fixed strain amplitude of 1% across a frequency range of 0.1–100 rad s^−1^ at 25 °C. The storage modulus (G') and loss modulus (G″) were recorded as functions of angular frequency to evaluate the viscoelastic behavior of the hydrogel system. The temperature was maintained constant throughout the measurement using the rheometer's built‐in temperature control system. All rheological measurements were performed in triplicate to ensure reproducibility. Data analysis and visualization were performed using GraphPad Prism software (version 9.0).

### Experimental Design and Treatment Model

All animal procedures were conducted in compliance with the Guide for the Care and Use of Laboratory Animals and approved by the Animal Ethics Committee of Fujian Medical University Union Hospital. Male C57BL/6 mice (8–10 weeks old, 20–25 g) were anesthetized with isoflurane (2–3%) and placed in the supine position. Following aseptic preparation, a midline laparotomy was performed with a 2‐cm incision. The cecum and adjacent small bowel were gently exteriorized and identified. Sterile gauze was folded into small pieces and used to create standardized serosal abrasions at the ileocecal junction to induce adhesive small bowel obstruction. The cecal surface and adjacent ileal segment were gently abraded with the folded gauze until a punctate hemorrhage appeared on the ileal wall near the ileocecal junction, indicating adequate serosal injury. Care was taken to avoid full‐thickness bowel wall injury or perforation. The abraded bowel segments were then exposed to room air for 5 min to promote peritoneal irritation and subsequent adhesion formation. The therapeutic intervention was initiated immediately after modeling (abrasion of the ileal wall near the ileocecal junction). Following treatment application, the bowel was gently returned to the peritoneal cavity. The abdominal wall was closed in two layers using 4–0 absorbable sutures for the peritoneum and muscle layers and 4–0 nonabsorbable sutures for the skin.

The animals were randomly divided into six groups (*n* = 10 per group): 1) a sham group, which underwent the surgical procedure without modeling;2) PBS group, which received 100 µL of PBS following adhesion model establishment; 3) the ASBO group (silk‐only), which received a topical application of the hydrogel vehicle following adhesion model establishment; ^(4)^ the BMSC@Gel group (silk hydrogel and BMSCs), which received a hydrogel containing BMSCs (3 × 10^6^ cells/100 µL) uniformly applied to the abraded serosal surface after modeling 5) the BMSCs monotherapy group (BMSC‐only), since free BMSCs cannot effectively adhere to the injured site after model establishment,^[^ ^72^ ^]^ this group received an equivalent number of BMSCs (3 × 10^7^ cells) via a tail vein injection to evaluate systemic effects and confirm the necessity of hydrogel‐based local delivery for cell retention and therapeutic efficacy; and 6) the prednisolone treatment group (conventional treatment group), which received immediate oral gavage of 1 mg kg^−1^ prednisolone dissolved in 0.2 mL of physiological saline following model establishment. The dosage was selected based on the effective doses reported for peritoneal adhesion prevention in rodent models.^[^ ^73^ ^]^ All surgical procedures were performed under aseptic conditions. Postoperatively, the animals received standardized care, including analgesic and prophylactic antimicrobial treatment. The animals were euthanized at designated time points (Days 1, 3, 5, and 7 after the operation), and intestinal specimens were harvested under sterile conditions.

For mechanistic studies, additional treatment groups were established: 7) TGF‐β pathway inhibition group: Following BMSC@Gel treatment, mice received intraperitoneal injection of TGF‐β inhibitor SB‐431542 (10 mg kg^−1^ dissolved in 5% DMSO solution) to evaluate the role of TGF‐β signaling in therapeutic efficacy. Beer BMSC@Gel group: BMSCs were pretreated with the neutral sphingomyelinase inhibitor GW4869 (10 µm, MCE Bioscience) for 24 h in exosome‐free serum medium to deplete exosome production. Cells were cultured to 80–90% confluence, and the final DMSO concentration was maintained below 0.01%. The exosome‐depleted BMSCs were then incorporated into silk fibroin hydrogels following the standard BMSC@Gel preparation protocol described in section 4.2.

### Mouse‐ and Human‐Derived Organoid Culture and CTCC‐E028‐MIC Cell Culture

Mouse intestinal crypts were isolated from the small intestine of 4–6 week‐old C57BL/6 mice. After cleaning and segmentation into 2‐mm pieces, the tissue was enzymatically digested at 4 °C for 30 min and then filtered through a 100‐µm mesh to obtain crypts for organoid culture using commercial growth medium. The pellet obtained after centrifugation was encapsulated in organoid culture ECM (BioGenous Biotechnology (Shanghai, China), Cat# M315066) and cultured in medium (BioGenous Biotechnology (Shanghai, China), Cat# K2001‐MI). Human‐derived organoids were cultured using the same method, with human tissue collection approved by the Clinical Sample Ethics Committee of Fujian Medical University Union Hospital (approval number: FJMU IACUC 2024‐Y‐2279).

### Immunohistochemistry and Immunofluorescence Staining

Intestinal tissues were fixed with 4% paraformaldehyde for 24 h at 4 °C and embedded in paraffin. Five‐micron‐thick sections were cut and mounted on glass slides. After deparaffinization and rehydration through a graded ethanol series, antigen retrieval was performed in citrate buffer (10 mm, pH 6.0) at 95 °C for 15 min. The sections were blocked with 5% bovine serum albumin (BSA) in phosphate‐buffered saline (PBS) for 1 h at room temperature to prevent nonspecific binding. Primary antibodies were applied and incubated overnight at 4 °C, followed by an incubation with appropriate fluorophore‐conjugated secondary antibodies for 1 h at room temperature. For immunohistochemistry (IHC), the DAB chromogen was used for visualization, followed by hematoxylin counterstaining; for immunofluorescence (IF) staining, fluorescently labeled secondary antibodies were applied, and the nuclei were counterstained with DAPI. Images were acquired using a digital slide scanner (KF‐PRO‐005/KF‐FL‐400, Jiangfeng Biology, China). The antibodies used in this study are listed in Table S2 (Supporting Information). For quantitative analysis, positive staining areas were measured using ImageJ software (Version 1.53, NIH, USA). The integrated optical density (IOD) and positive area percentages were calculated from at least five randomly selected fields per section at 200 × magnification. Consistent imaging parameters (exposure time, gain settings) were maintained across all samples to ensure standardized quantification. Results were expressed as mean ± standard deviation from multiple biological replicates (*n* = 10 biologically independent samples per group), with statistical significance determined at ^*^ *p* < 0.05 and ^**^ *p* < 0.01.

### Histological Analysis

Intestinal tissue samples were fixed, embedded in paraffin, and sectioned at a thickness of 5 µm. For the morphological evaluation, hematoxylin and eosin (H&E) staining was performed to assess tissue injury, whereas Masson's trichrome staining was used to evaluate the severity of fibrosis. Two experienced pathologists, who were blinded to the experimental groups, independently scored six random fields per sample. The scoring criteria are presented in Table S1 (Supporting Information), and the data are presented as median scores with interquartile ranges.

### Proteomic and Bioinformatic Analyses

Exosomal proteins were isolated through differential ultracentrifugation and chromatographic purification, characterized using multiple analytical methods, processed for the LC‒MS/MS analysis, and subjected to comprehensive bioinformatic analyses including protein identification, differential expression, pathway analysis, and interaction network construction.

Functional enrichment analyses were performed using the Gene Ontology (GO) and Kyoto Encyclopedia of Genes and Genomes (KEGG) databases to identify significantly enriched biological functions and pathways. For the GO analysis, the focus was placed on biological process (BP) terms using the complete GO annotation database. Differentially expressed proteins were identified using the following criteria: log_2_|FC| > 1.2 and *p* < 0.05. The phyper function in R software was used to calculate *p* values based on hypergeometric distributions, which were subsequently adjusted for multiple testing using the Benjamini–Hochberg false discovery rate (FDR) correction to obtain Q values. GO terms and KEGG pathways with Q values ≤ 0.05 and containing at least 3 genes were considered significantly enriched. Background gene sets were defined as all proteins detected in theLC‒MS/MS analysis.

Gene set enrichment analysis (GSEA) was performed to identify coordinated changes in predefined gene sets using the fgsea package in R. GSEA was conducted using ranked gene lists based on log_2_|FC| values. Normalized enrichment scores (NESs) were calculated to measure the degree of enrichment of gene sets at the top or bottom of the ranked list. Gene sets with |NES| > 1.5 and FDR < 0.05 were considered significantly enriched. KEGG and GO gene sets from the Molecular Signatures Database (MSigDB) were used for the analysis. Log_2_|FC| indicates the log_2_ fold change.

### Isolation and Characterization of Exosomes

Exosomes were isolated according to the manufacturer's instructions (Umibio, China). For exosome isolation and purification, the cell culture supernatant (≥20 mL) was thawed in a 25 °C water bath if frozen or placed on ice if fresh. The supernatant was centrifuged at 3000 × g (≈5,200 rpm) for 10 min at 4 °C to remove cellular debris, followed by centrifugation at 10 000 × g (≈9,500 rpm) for 10 min at 4 °C to remove impurities. The cleared supernatant was mixed with Exosome Concentration Solution according to the manufacturer's protocol and incubated at 4 °C for 8–24 h. After the incubation, the mixture was centrifuged at 10 000 × g for 60 min at 4 °C, followed by an additional 2 min centrifugation at the same speed to obtain the exosome pellet. The pellet was resuspended in PBS (≈200 µL of PBS per 20 mL of the initial supernatant) and centrifuged at 12 000 × g (≈12 400 rpm) for 2 min at 4 °C. The supernatant containing the exosomes was purified using an exosome purification filter by centrifugation at 3000 × g (≈6,200 rpm) for 10 min at 4 °C. The purified exosomes were aliquoted and stored at −80 °C until further analysis. The samples were observed under an electron microscope to determine whether the exosomes exhibited a typical cup‐shaped morphology. Western blot analysis was performed to examine the characteristic expression of exosomal markers TSG101, CD9, CD81, CD63, and Alix, while confirming the absence of the cellular contaminant marker GM130, thereby validating successful exosome purification and purity. Additionally, nanoparticle tracking analysis (NTA) was employed to verify that the exosome diameter met standard size criteria.

### Single‐Cell Sequencing

The scRNA‐seq analysis was performed using the BD Rhapsody WTA Analysis Pipeline (version 1.8) with FASTQ files, reference genome files (mouse: mm10), and transcriptome annotation files (mouse: GENCODE vM23/Ensembl 98) used for the sequence alignment. The resulting unique molecular identifier (UMI) count matrix was analyzed using the Python package Scanpy (version 1.8). For quality control in microdroplet‐based experiments, cells with UMI counts ranging from 500 to 60 000, detected gene numbers ranging from 200 to 8000, and a mitochondrial gene content ≤60% were included, resulting in 43 704 cells (BMSC@Gel) and 42 275 cells (adhesion model) for the downstream analysis.^[^ ^74^ ^]^

For mouse fibroblast analysis, more stringent filtering criteria were applied: cells with mitochondrial gene content <20%, detected gene numbers ranging from 200 to 7000 were retained for further analysis. Library size normalization was performed using the pp.normalize_total function, where gene expression measurements were normalized to the total expression, scaled by 10000, and log‐transformed using the pp.log1p function. Highly variable genes were identified using Macosko's method with the pp.highly_variable_genes function, with 3000 highly variable genes selected for downstream analysis.

Batch correction was performed using Harmony with sample_name as the batch key to remove potential batch effects between samples. Dimensionality reduction was achieved through principal component analysis (PCA) ^[^ ^75^ ^]^ using the tl.pca function with 50 principal components, followed by graph‐based cell clustering using the pp.neighbors function. Cell clustering was performed using the Leiden algorithm with a resolution of 1.0. The cells were visualized in both 2D and 3D using uniform manifold approximation and projection (UMAP) with the tl.umap function. Cluster‐specific marker genes were identified using the FindAllMarkers function (the Wilcoxon test was used) in Seurat, with significance defined as an adjusted *p*‐values < 0.05 and |log2foldchange| > 0.25. The functional enrichment analysis of the marker genes was conducted using the R package ClusterProfiler, and a hypergeometric test was used to evaluate the enrichment of biological processes, including Gene Ontology (GO) terms and KEGG pathways.

### Spatial Transcriptomics and Cell‐Cell Communication Analysis Sample Preparation and Sequencing

Fresh tissue samples were rapidly processed for spatial transcriptomics analysis using the 10x Visium platform. Tissues were quickly rinsed with pre‐cooled PBS, dried with sterile gauze, and immediately submerged in isopentane for ≈1 min until completely frozen. Samples were then embedded in OCT compound, ensuring bubble‐free embedding, and stored at −80 °C until sectioning.

Frozen sections (10–20 µm thickness) were prepared for RNA quality assessment using RIN evaluation and H&E staining for morphological verification. Only samples with RIN ≥ 7 and intact histomorphology proceeded to subsequent analysis. Tissue optimization was performed using the 10x Genomics tissue optimization kit (CG000240) to determine optimal permeabilization conditions specific to this tissue type. Following optimization, formal spatial capture experiments were conducted, including tissue placement, fixation, H&E staining, permeabilization, cDNA synthesis, amplification, and library construction. Quality‐controlled libraries were sequenced using the Illumina NovaSeq PE150 strategy.^[^ ^76^ ^]^

To decipher intercellular communication networks, CellChat analysis was employed using the R package with the default CellChatDB database. Signaling pathways were identified, communication probabilities were calculated, and comprehensive communication networks were constructed to determine ligand‐receptor pair relationships among different cell types. Communication probabilities were computed at both individual ligand‐receptor and signaling pathway levels, visualized through Hierarchy and Circle plots. Network centrality scores identified key signals contributing to outgoing/incoming signaling, with pattern inference performed using the NMF R package and visualized via river plots showing associations between latent patterns, cell groups, and signaling pathways.

### Nair Adhesion Scoring System

Adhesion severity was assessed using the validated Nair adhesion grading scale, which classifies adhesions into five grades based on macroscopic characteristics: Grade 0 (no adhesion, freely mobile tissues), Grade 1 (thin, avascular adhesions amenable to blunt dissection), Grade 2 (thicker, partially vascularized adhesions requiring blunt dissection with minimal bleeding), Grade 3 (dense, vascularized fibrous bands requiring sharp dissection), and Grade 4 (complete tissue fusion precluding safe separation). All adhesion scores were normalized to the Day 1 BMSC@Gel mean values (set as 1.0) to enable standardized intergroup comparisons. Adhesion severity was evaluated using the Nair grading system (0–4).

Data are presented as median (interquartile range) and frequency distributions. Between‐group comparisons were performed using Mann–Whitney U test or Kruskal–Wallis test with Dunn's post‐hoc analysis. Animals were randomly allocated to treatment groups using stratified randomization by body weight (*n* = 10 per group). Statistical significance was set at *p* < 0.05.

### Multiplex Immunofluorescence Analysis

Multiplex immunofluorescence staining was performed using a Five‐color mIHC Fluorescence Kit Plus (Huilanbio Biological Technology, Shanghai, China) based on tyramide signal amplification (TSA) technology according to the manufacturer's instructions. Briefly, paraffin‐embedded tissue sections (4 µm) were deparaffinized, rehydrated, and subjected to antigen retrieval in EDTA buffer (pH 9.0) with microwave heating. After blocking endogenous peroxidase activity and nonspecific binding, the sections were sequentially incubated with primary antibodies against VIMENTIN, PDGFRA, TOP2A, and IGFBP3 overnight at 4 °C, followed by an incubation with an HRP‐conjugated secondary antibody for 50 min at room temperature. TSA fluorescent dyes were applied for signal amplification (1:200 dilution, 10 min), and the antibody complexes were stripped between rounds by heating at 95 °C for 25–40 min. The nuclei were counterstained with DAPI, and the sections were mounted with antifade medium for the fluorescence microscopy examination. PDGFRA+TOP2A+ cells were identified as proliferating FBs, whereas PDGFRA+IGFBP3+ cells were identified as inflammatory FBs.

Moreover, the expression of COL4A2, a major structural component of the basement membrane, and COL6A1 and COL6A2, which play crucial roles in aberrant tissue repair processes and scar formation, was also examined. The antibodies used in this study are listed in Table S2 (Supporting Information).

### Reactive Oxygen Species Detection

Intracellular ROS levels were measured using DCFH‐DA (Reactive Oxygen Species Assay Kit, Beyotime Biotechnology, China) at the designated time points. BMSCs released from hydrogels (BMSC@Gel), monocultured control BMSCs, and positive control ROS(+) cells were analyzed according to the manufacturer's optimized protocol. The DCFH‐DA stock solution was diluted 1:1000 in serum‐free medium to achieve a final working concentration of 10 µm. The culture medium was removed and replaced with the diluted DCFH‐DA solution, followed by an incubation at 37 °C for 20 min with gentle mixing every 3–5 min. The cells were subsequently washed three times with PBS to remove the unincorporated probe. For the positive control, the cells were treated with Rosup (50 µg mL^−1^) for 20–30 min to induce oxidative stress. The fluorescence intensity was measured using a laser scanning confocal microscope with excitation at 488 nm and emission at 525 nm, with the background fluorescence subtracted from that of unstained control cells. The ROS levels were quantified as relative fluorescence units, with a higher fluorescence intensity indicating elevated oxidative stress.

### Analysis of the Mitochondrial Membrane Potential

The mitochondrial membrane potential was evaluated using a JC‐1 staining kit (Beyotime Biotechnology, China) according to the manufacturer's protocol. The JC‐1 working solution was prepared by diluting the JC‐1 stock solution (200X) with ultrapure water at a ratio of 50 µL stock to 8 mL water, followed by vigorous vortexing to ensure complete dissolution, and then adding 2 mL of JC‐1 staining buffer (5X) to achieve the final working concentration. For the positive control, the cells were pretreated with carbonyl cyanide 3‐chlorophenylhydrazone (CCCP, 10 µm) for 20 min to induce the loss of the mitochondrial membrane potential. For staining, the culture medium was removed from the cells and replaced with 1 mL of fresh culture medium, followed by the addition of 1 mL of JC‐1 working solution with thorough mixing. The cells were incubated at 37 °C for 20 min and then washed twice with prechilled JC‐1 staining buffer (1X) prepared by diluting the 5X buffer 1:4 with distilled water. After washing, 2 mL of fresh culture medium was added, and the fluorescence was immediately observed using a fluorescence microscope. A high mitochondrial membrane potential was indicated by red fluorescence (JC‐1 aggregates), whereas a low membrane potential was indicated by green fluorescence (JC‐1 monomers). The ratio of red to green fluorescence intensities was calculated to quantitatively assess changes in the mitochondrial membrane potential, with CCCP‐treated cells serving as positive controls for membrane potential disruption.

### Quantification and Statistical Analysis

All the data are presented as the means ± SEM, and groups were compared using Student's *t* test. Differences were considered statistically significant at ^*^ *p* < 0.05, ^**^ *p* < 0.01, and ^***^ *p* < 0.001. In vitro experiments, such as CCK‐8, Western blotting, and wound healing assays, were routinely repeated at least three times. The animals were randomly grouped based on body weight. Differentially expressed genes among the subpopulations were examined via Gene Ontology (GO) analysis, Kyoto Encyclopedia of Genes and Genomes (KEGG) analysis, and gene set enrichment analysis (GSEA) using the default methods in the corresponding software packages. Significant pathway activity was determined through random permutation tests. Student's *t* test was used to analyze differences in cell frequencies and expression levels between the ASBO group and posttreatment samples.

### Ethical Approval Statement

All human tissues were collected after obtaining written informed consent from patients, and the procedures were conducted in accordance with the principles of the Declaration of Helsinki. The study was approved by the Clinical Sample Ethics Committee of Fujian Medical University Union Hospital (approval number: FJMU IACUC 2024‐Y‐2279). All animal procedures were performed in accordance with the Guide for the Care and Use of Laboratory Animals and were approved by the Institutional Animal Care and Use Committee (IACUC) of Fujian Medical University Union Hospital (approval number: FJMU IACUC 2024‐Y‐2279).

## Conflict of Interest

The authors declare no conflict of interest.

## Author Contributions

L. Z. and J.Z., along with X.C., were responsible for the conceptualization of the study. L.Z. J.Z., and Z.H. are co‐first authors and contributed equally to this work. P.H. handled the methodology, whereas L.Z. and Z.L. conducted the investigation. P.H. and J.Z. worked on visualization. X.C. secured funding for the project. Z.H. and L.Z.were responsible for project administration, with J.Z. and D.Z. providing supervision. X.C. performed the review and editing of the article.

## Supporting information



Supporting Information

## Data Availability

Research data are not shared.
