# Supplementary material for: Bone Marrow Mesenchymal Stem Cell Hydrogel‐Mediated Fibroblast Reprogramming Restores Intestinal Function in Adhesive Small Bowel Obstruction
Source: Adv Sci (Weinh). 2025 Oct 27;13(3):e13781. doi: 10.1002/advs.202513781 (PMC12806514; doi:10.1002/advs.202513781)
Supplement: Supplementary file 1 — Supporting Information [file ADVS-13-e13781-s001.docx]

**Bone Marrow Mesenchymal Stem Cell Hydrogel-Mediated Fibroblast Reprogramming Restores Intestinal Function in Adhesive Small Bowel Obstruction**

**Authors**

*Lihong Zheng 1,2†, Junrong Zhang 1,2†, Zhengyuan Huang 1,2†, Zhenliang Lin 1,2, Jin Zhang 2, Da Zhang4***, Ping Hou3**, Xianqiang Chen 1, 2**

**Figure S1**

**
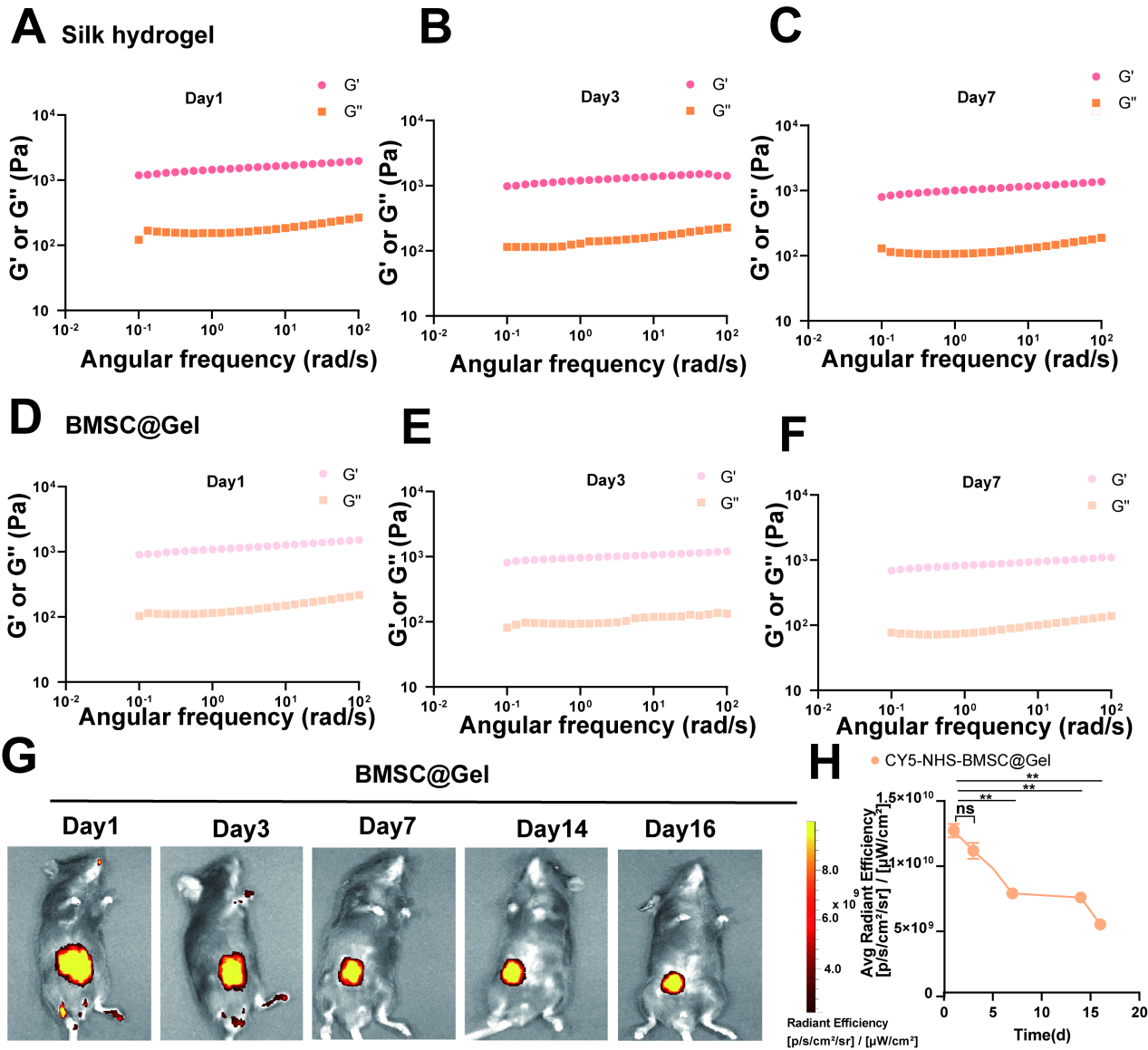
**

**Figure 1. Rheological characterization and in vivo tracking of silk fibroin hydrogel and BMSC@Gel system.** (A-C) Storage modulus (G') and loss modulus (G'') of silk fibroin hydrogels as functions of angular frequency on Days 1, 3, and 7. (D-F) Rheological properties of BMSC@Gel showing similar viscoelastic behavior over 7 days. Both systems maintained stable moduli around 1 kPa. (G) Representative bioluminescence images show the distribution and persistence of the CY5-NHS-BMSC@Gel implant from Day 1 to Day 16 post-operation. (H) Quantitative analysis of CY5-NHS-BMSC@Gel signal intensity over time (n=3), *P < 0.05, **P < 0.01 vs. Day 1.

**Figure S2**

**
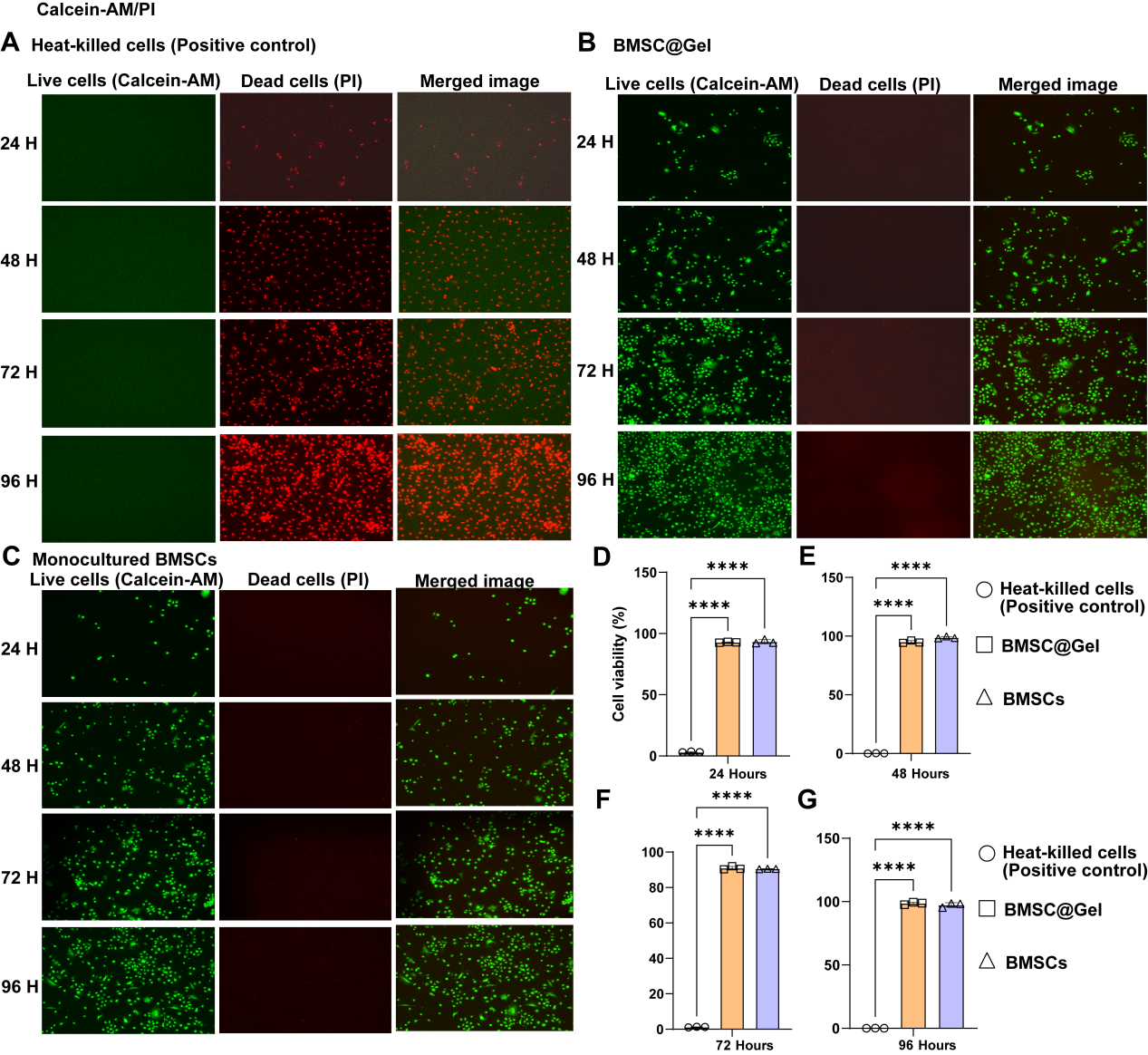
**

**Figure S2. Assessment of BMSC viability during release from the hydrogel.** (A-C) Calcein-AM/PI staining showing live (green) and dead (red) cells over 96 hours for heat-killed cells (A), BMSC@Gel (B) and monocultured BMSCs (C). (D-G) Quantitative viability analysis demonstrating >90% cell survival in the BMSC@Gel and monocultured groups compared with the heat-killed control groups (p<0.0001) at 24 hours (D), 48 hours (E), 72 hours (F), and 96 hours (G). Significant differences between viable cell groups (BMSC@Gel and monocultured BMSCs) compared with heat-killed controls at all time points (p<0.0001). Data are presented as means ± SEM, (n=3 independent experiments).

**Figure S3**

**
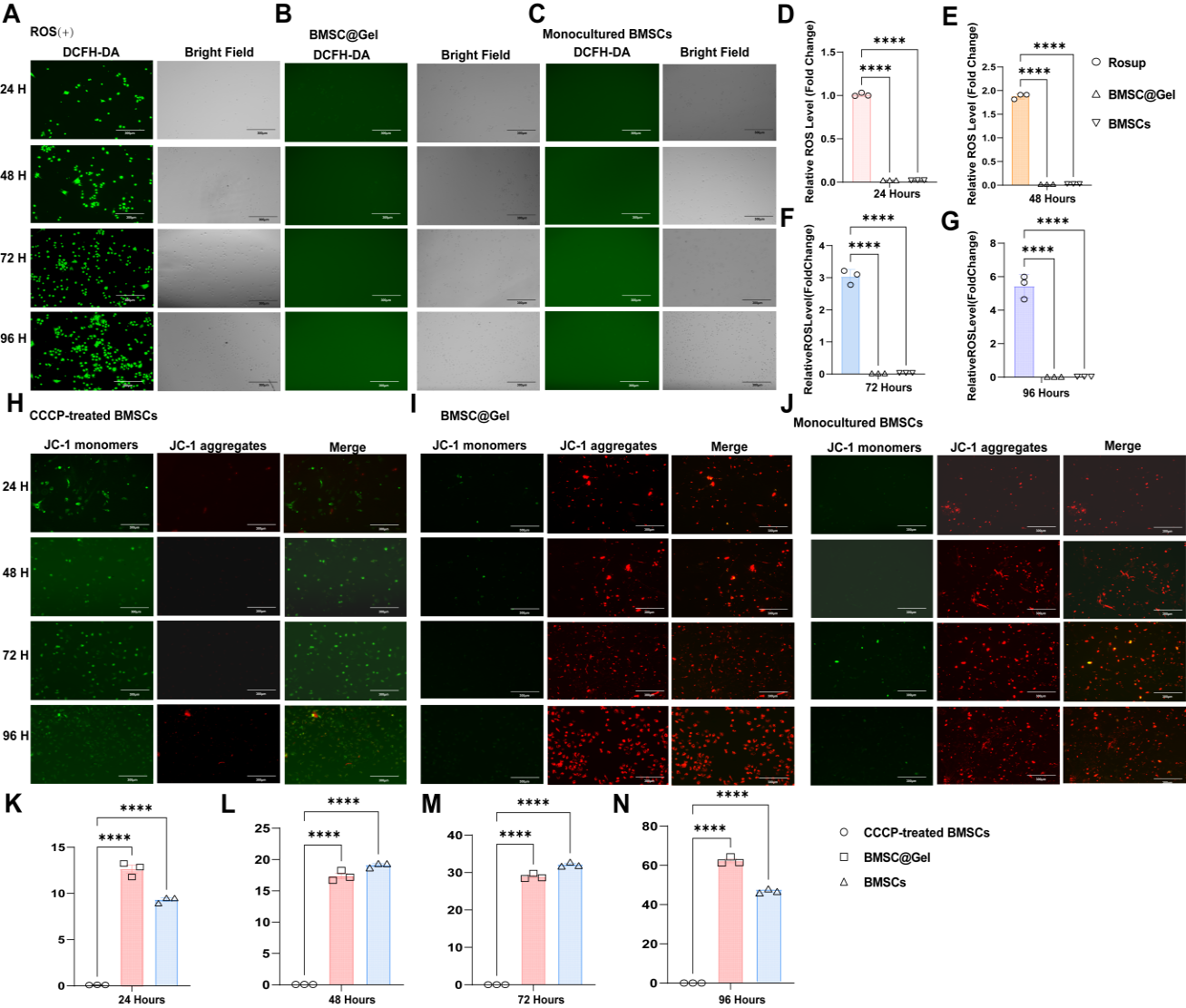
**

**Figure S3. Analyses of ROS levels and the mitochondrial membrane potential of the BMSCs released from the hydrogels.** (A-C) DCFH-DA staining for intracellular ROS detection: hydrogel-released BMSCs and monocultured BMSCs maintained low ROS levels and presented weak green fluorescence, whereas the ROS-up-treated positive controls (+) presented bright green fluorescence, indicating significant ROS production. (D-G) Quantitative analysis of intracellular reactive oxygen species (ROS) levels in different experimental groups at 24 hours (D), 48 hours (E), 72 hours (F), and 96 hours (G). The ROS-up group (positive control for oxidative stress) demonstrated significantly elevated ROS levels compared to BMSC@Gel and BMSC groups. Significant differences between the ROS-up group and both BMSC treatment groups at all time points (p < 0.0001). No significant differences were observed between BMSC@Gel and BMSC groups (p > 0.05). Data are presented as means ± SEM. (H-J) JC-1 staining for mitochondrial membrane potential assessment: hydrogel-released BMSCs preserved mitochondrial membrane potential and displayed red fluorescence (JC-1 aggregates), which was comparable to that of monocultured BMSCs, whereas carbonyl cyanide 3-chlorophenylhydrazone (CCCP)-treated positive controls presented green fluorescence (JC-1 monomers), indicating loss of mitochondrial membrane potential. (K-N) Quantitative analysis revealed significant differences between CCCP-treated positive controls and both BMSC treatment groups at all time points (p < 0.0001). Data are presented as means ± SEM, (n=3).

**Figure S4**

**
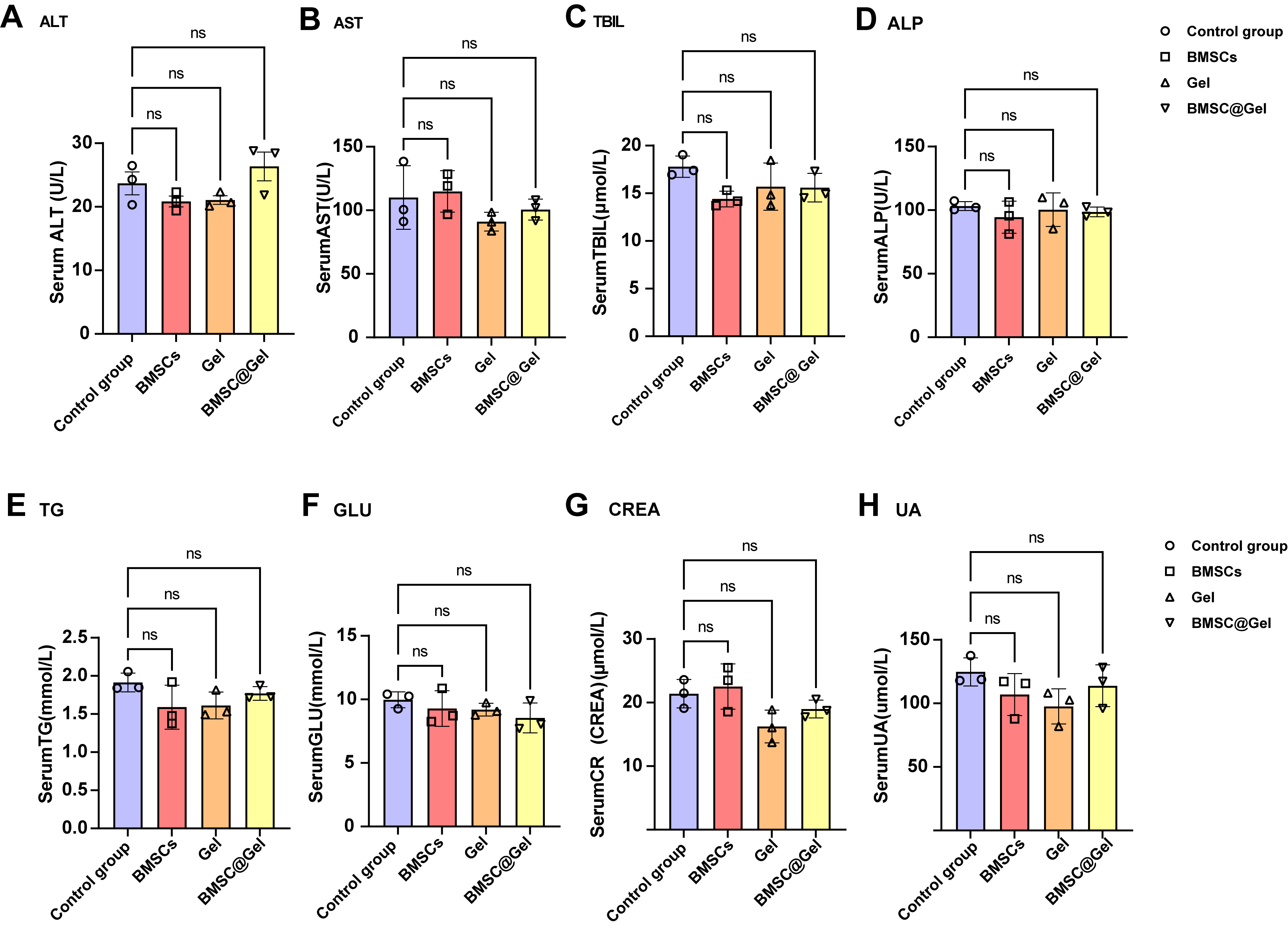
**

**Figure S4. Systemic safety assessment via a serum biochemical analysis.** (A-H) Liver function (ALT, AST, TBIL, and ALP), metabolic (TG and GLU), and kidney function (CREA, UA) marker levels did not differ significantly among the treatment groups (ns, not significant). The data are presented as the means ± SEM, (n=3).

**Figure S5**

**
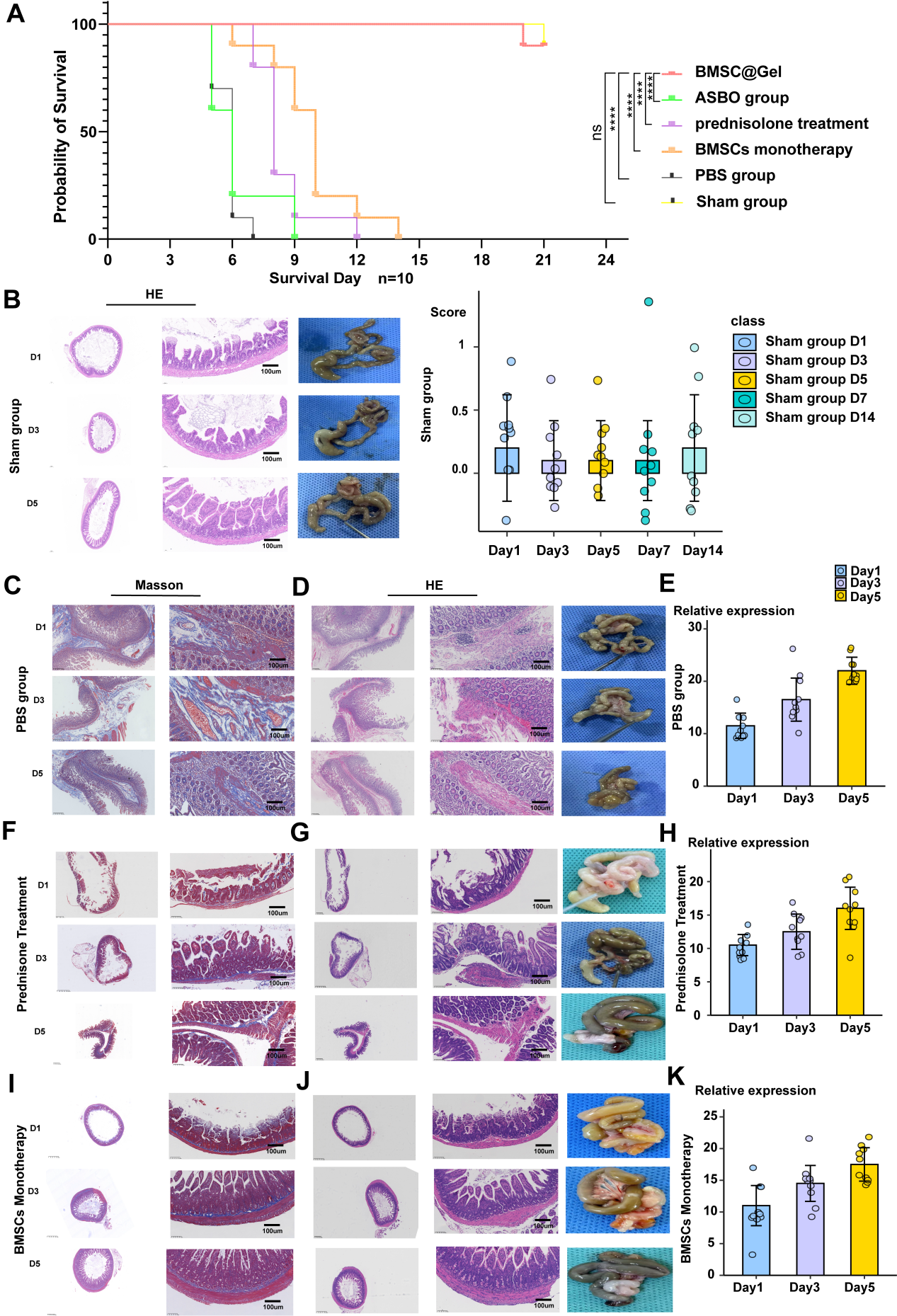
**

**Figure S5. Histopathological analysis and survival outcomes of the treatment groups.** (A) Kaplan-Meier survival analysis of six experimental groups: Sham, PBS, prednisolone treatment, BMSC monotherapy, ASBO (hydrogel alone), and BMSC@Gel groups (n=10 per group). Log-rank test showed significant differences among groups (χ²=55.86, df=5, p<0.0001). The BMSC@Gel group achieved survival rates comparable to the Sham group, while the ASBO group demonstrated the highest mortality (75-80% by Day 7). (B) H&E staining and Nair scoring confirmed that the sham group (with laparotomy alone) showed no significant adhesion formation. Scale bar = 50 μm. (C-E) PBS control group. (C) Masson's trichrome staining showed extensive collagen deposition (blue) and disrupted tissue architecture. (D) H&E staining revealed severe mucosal damage with inflammatory cell infiltration and villous disorganization. The gross samples showed dense adhesion band formation between intestinal loops. (E) Nair adhesion scores over time. Scale bar = 100 μm. (F-H) Prednisolone treatment group. (F) Masson's trichrome staining showed persistent collagen deposition in the intestinal tissues. (G) H&E staining showed extensive inflammatory cell infiltration with adhesion band formation. (H) Nair adhesion scores over time. Scale bar = 100 μm. (I-K) BMSC monotherapy group. (I) Masson's trichrome staining revealed a partial reduction in collagen accumulation with limited improvement in tissue architecture. (J) H&E staining showed slight mucosal improvement with incomplete resolution of inflammatory changes. Gross samples showed partial reduction in adhesion formation with persistent obstruction. (K) Nair adhesion scores over time. Scale bar = 100 μm. **Statistical analysis:** All adhesion scores were normalized to Day 1 sham group values (set as 1.0). p < 0.0001 for all treatment groups vs. Sham group comparisons across all time points.

**Figure S6**

**
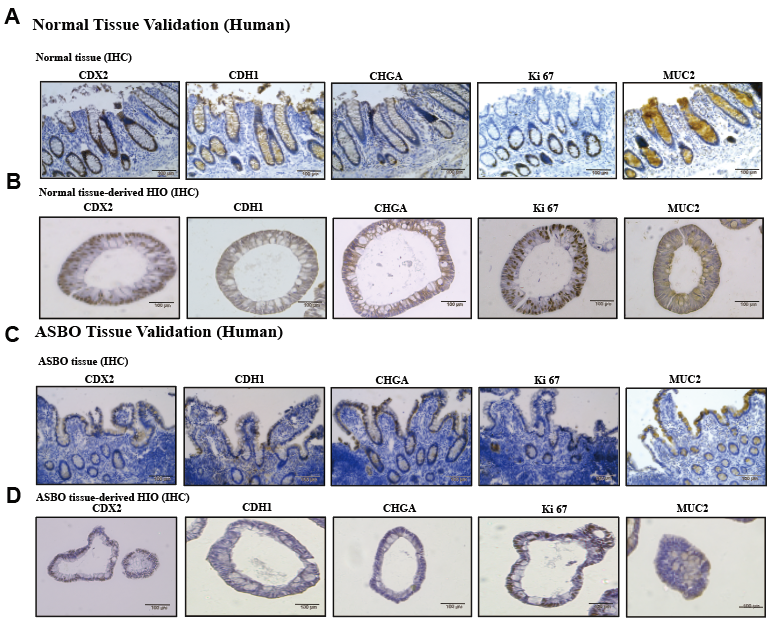
**

**Figure S6. Altered expression of intestinal markers in adhesive small bowel obstruction (ASBO) patients compared to normal controls. (**A-B) Immunohistochemical analysis showing differential expression patterns of intestinal markers (CDX2 and CDH1), endocrine marker (CHGA), proliferation marker (Ki67), and mucin protein (MUC2) in normal human intestinal tissue versus ASBO patient tissue. Normal tissue and corresponding intestinal organoids exhibit higher expression levels of all examined markers compared to ASBO samples. (C-D) Consistent downregulation of these markers is observed in both ASBO patient tissue and corresponding intestinal organoids. Scale bar = 100 μm.

**Figure S7**

**
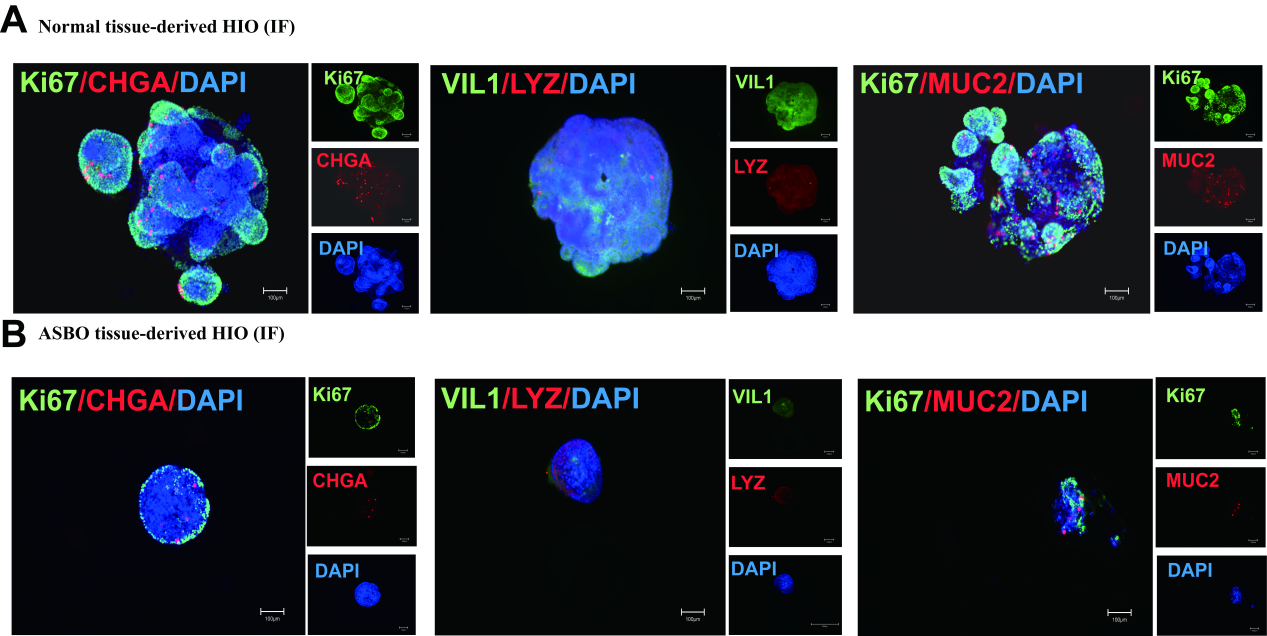
**

**Figure S7. Reduced expression of intestinal functional markers in ASBO patient-derived organoids.** (A-B) Immunofluorescence analysis of intestinal organoids derived from normal human tissue versus ASBO patient tissue, showing expression patterns of mucosal integrity markers (VIL1), enteroendocrine marker (CHGA), proliferation marker (Ki67), aneth cell marker (LYZ), and mucin secretion marker (MUC2). Fluorescence intensity of all examined markers was significantly lower in ASBO patient-derived organoids compared to organoids established from normal control tissue. Scale bar = 100 μm.

**Figure S8**


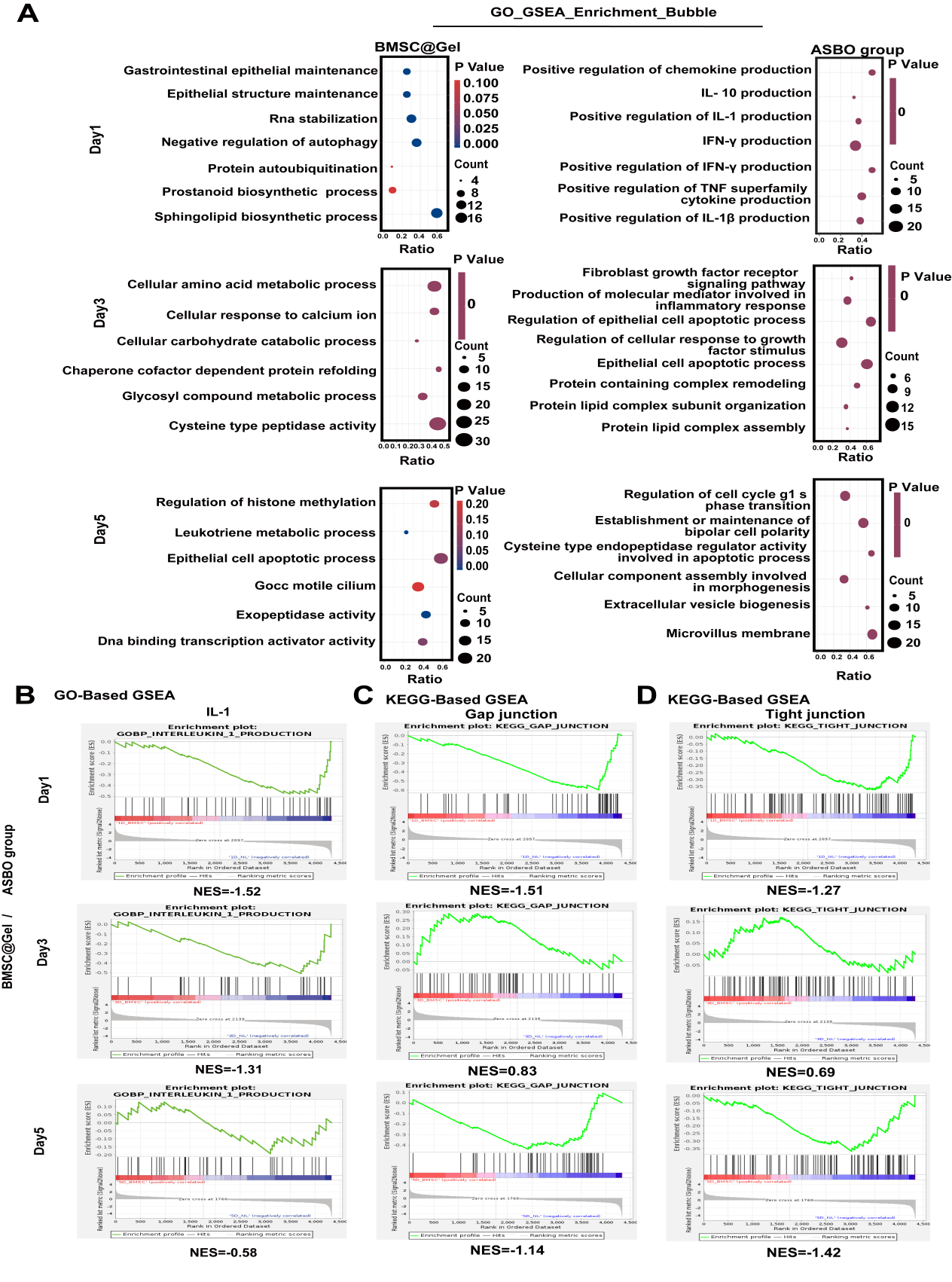


**Figure S8. GSEA of key cellular pathways in the BMSC@Gel and ASBO groups at different time points.** (A-B) The GSEA-GO analysis comparing the BMSC@Gel group with the ASBO group revealed that IL-1 was significantly inhibited in the BMSC@Gel group, while proinflammatory pathways were enriched in the ASBO group. (C) Gap junction pathway: GSEA enrichment plots showing temporal changes on Day 1 (top panel, NES=-1.51), Day 3 (middle panel, NES=0.83), and Day 5 (bottom panel, NES=-1.14) between the BMSC@Gel group and the ASBO group. (D) Tight junction pathway: GSEA enrichment plots showing the temporal progression on Day 1 (top panel, NES=-1.27), Day 3 (middle panel, NES=0.69), and Day 5 (bottom panel, NES=-1.42).

**Figure S9**

**
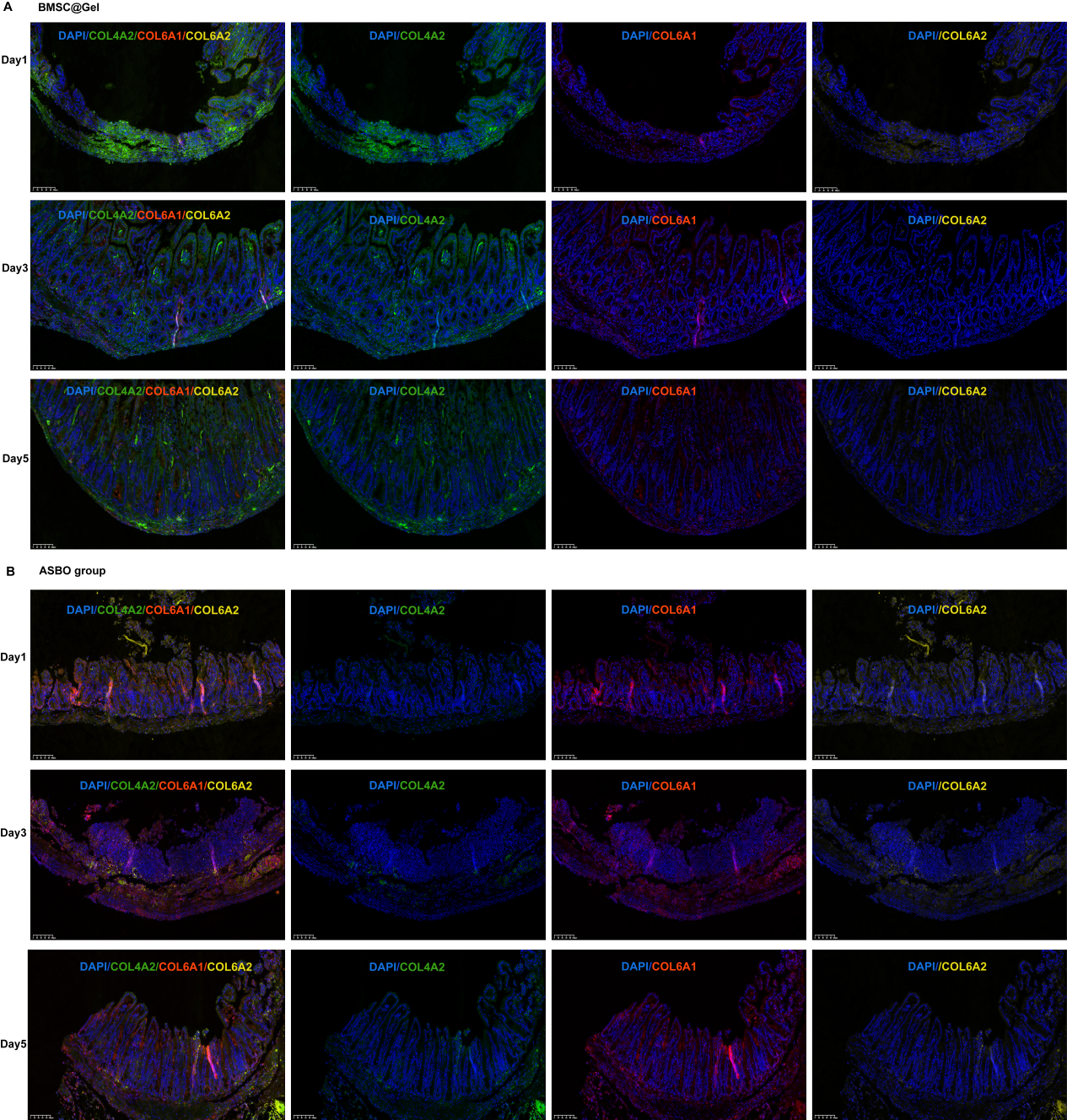
**

**Figure S9. Immunofluorescence analysis of the expression of different types of collagens in the ASBO and BMSC@Gel groups.** (A) BMSC@Gel group. Representative images of immunofluorescence staining showing the expression of COL4A2 (green), COL6A1 (red), and COL6A2 (yellow) on Days 1, 3, and 5 postsurgery. DAPI (blue) indicates the cell nuclei. Scale bar = 100 μm. (B) ASBO group. Representative images of immunofluorescence staining showing the expression of COL4A2 (green), COL6A1 (red), and COL6A2 (yellow) on Days 1, 3, and 5 postsurgery. DAPI (blue) indicates the cell nuclei. Scale bar = 100 μm. The BMSC@Gel group presented high expression of COL4A2, whereas the ASBO group presented elevated expression of COL6A1 and COL6A2, indicating distinct collagen deposition patterns between the treatment groups during adhesion formation.

**Figure S10**


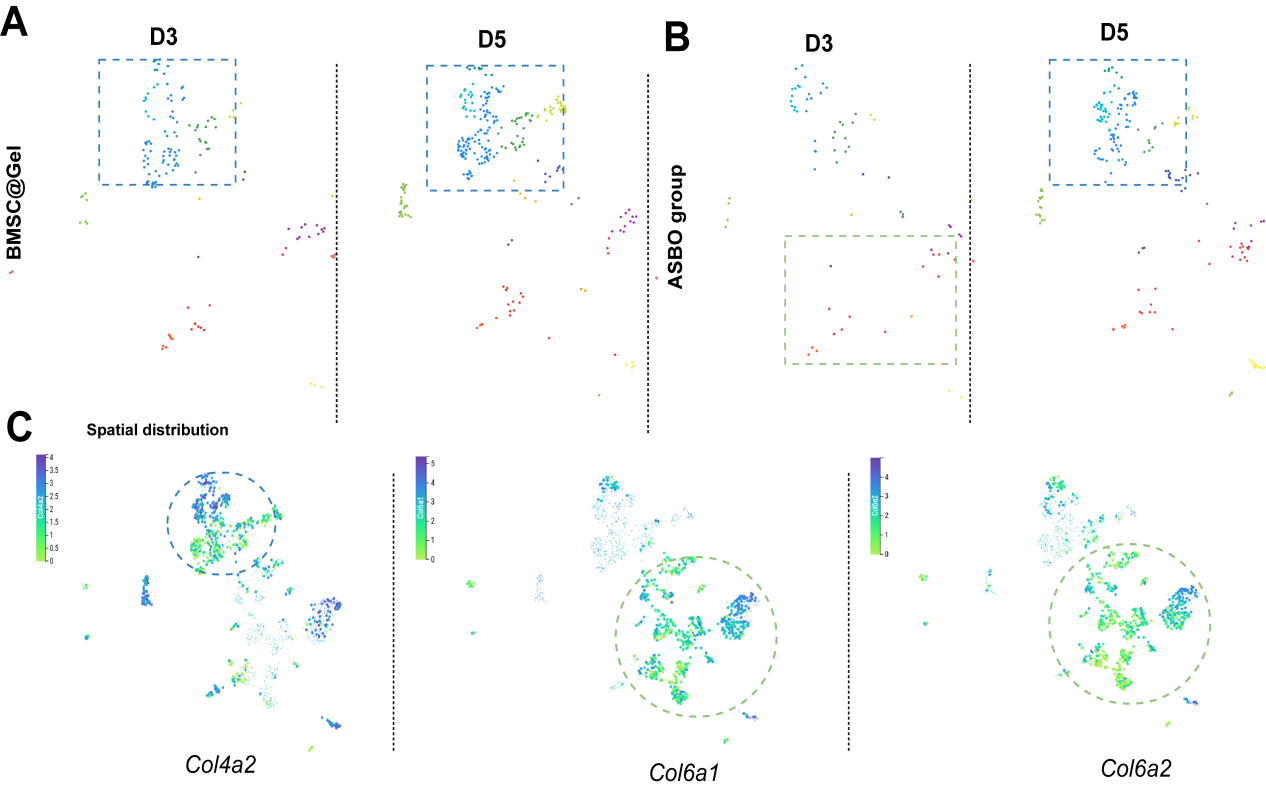


**Figure S10. Single-cell transcriptomic analysis of fibroblast dynamics.**(A and B) Spatiotemporal profile of fibroblast populations showing the enrichment of Proliferating FBs in the BMSC@Gel group compared with the temporal transition from inflammatory to proliferating FBs in the ASBO group by Day 5. (C) Spatial distribution of ECM signature genes (*Col4a2, Col6a1*, and *Col6a2*).

**Figure S11**


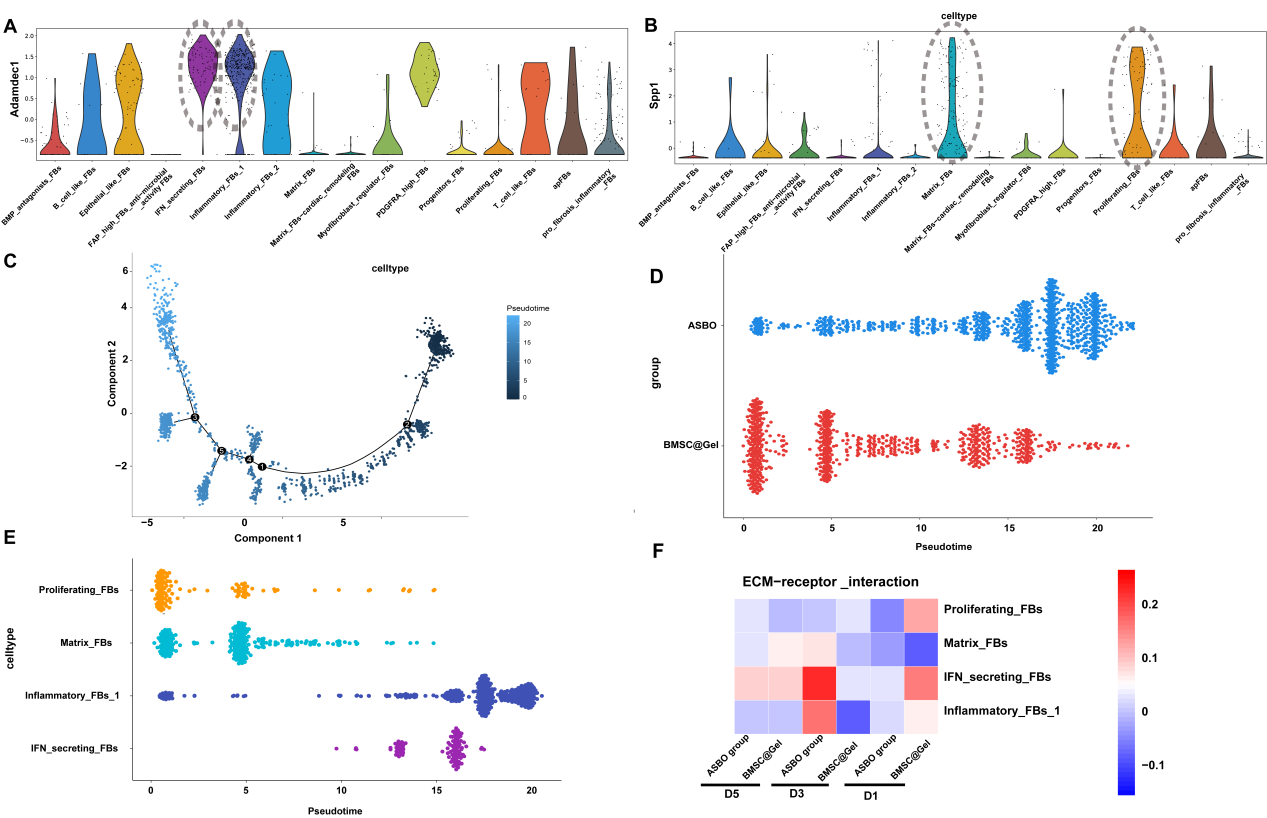


**Figure 11. Pseudotime trajectory analysis of fibroblast differentiation.** (A-B) Violin plots showing fibroblast-associated gene expression across different cell populations in the ASBO and BMSC@Gel groups. Adamdec1 (a biomarker of inflammatory FB-1s) was enriched in IFN-secreting FBs, whereas Spp1 (a biomarker of proliferating fibroblast marker) was also enriched in matrix FBs. Both IFN-secreting FBs and matrix FBs are defined as terminal differentiation states. (C-E) The pseudotime trajectory analysis revealed distinct fibroblast differentiation pathways between the ASBO and BMSC@Gel groups. The FBs in the ASBOs advanced into pathological phenotypes at the late stage, whereas the FBs in the BMSC@Gel group remained at the early stage of the differentiation trajectory. (F) Heatmap showing that the ECM‒receptor interaction pathway was further enriched between different fibroblast subpopulations.

**Figure S12**

**
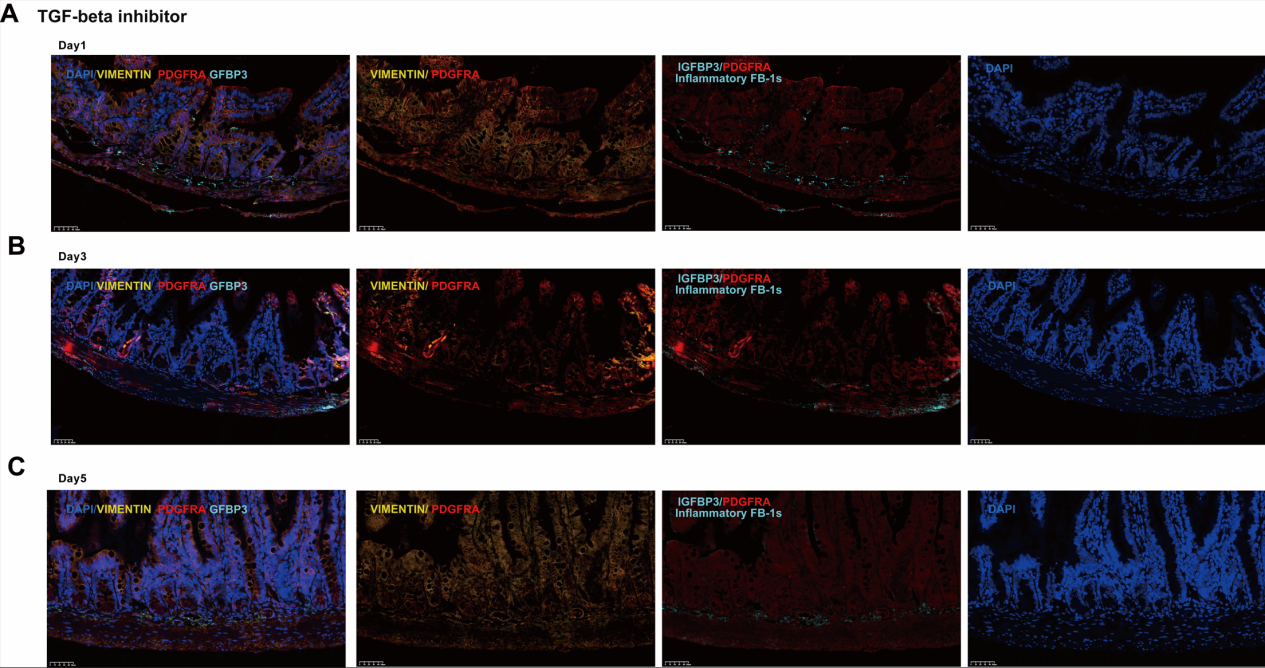
**

**Figure S12. TGF-β pathway inhibition significantly suppressed inflammatory fibroblast phenotypes.** (A-C) Immunofluorescence analysis of fibroblast phenotypes following TGF-β pathway inhibition on Days 1, 3, and 5 post-treatment compared to control. Progressive suppression of inflammatory fibroblast markers was observed with sustained inhibition. Scale bar = 100 μm.

**Figure S13**

**
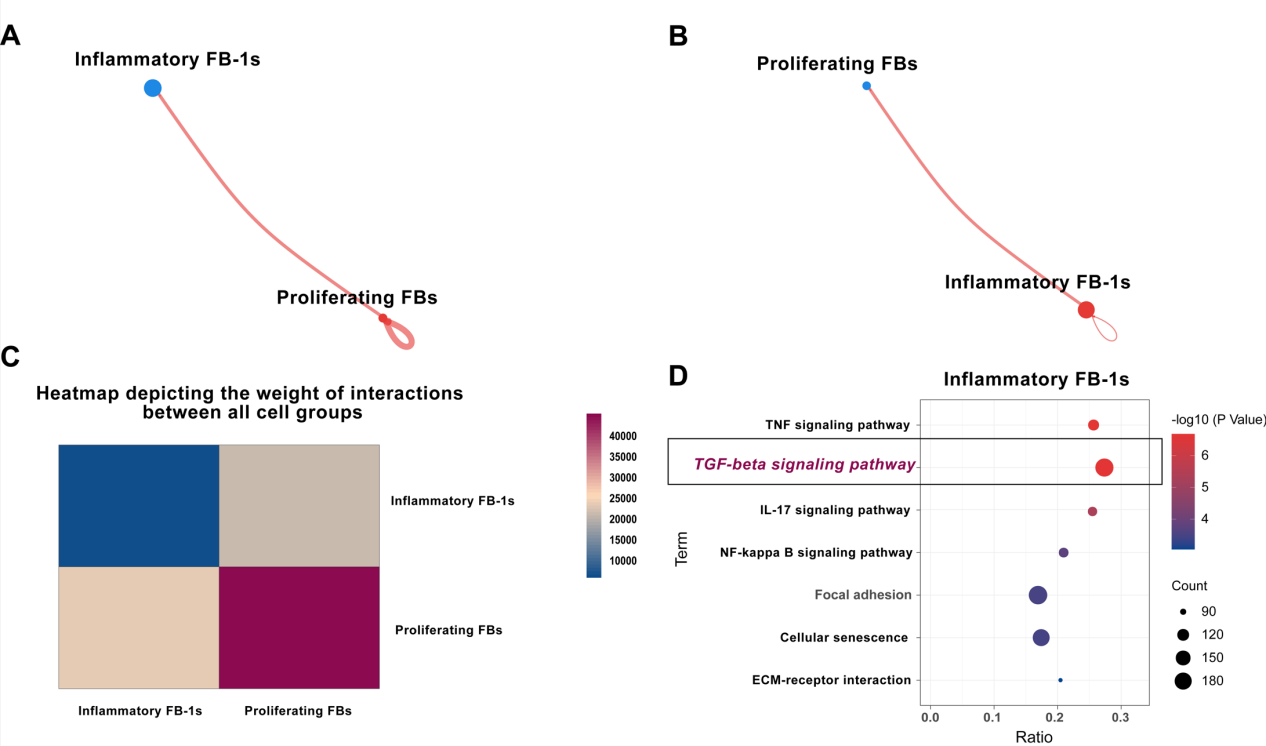
**

**Figure S13. Intercellular interactions and molecular mechanisms between inflammatory and proliferative fibroblasts.** (A-B) CellChat analysis reveals bidirectional communication networks between the two fibroblast subpopulations. (C) Interaction intensity heatmap quantifying intercellular communication strength. (D) KEGG enrichment analysis revealed that TGF-β signaling pathways were significantly enriched in inflammatory fibroblasts.

**Figure S14**


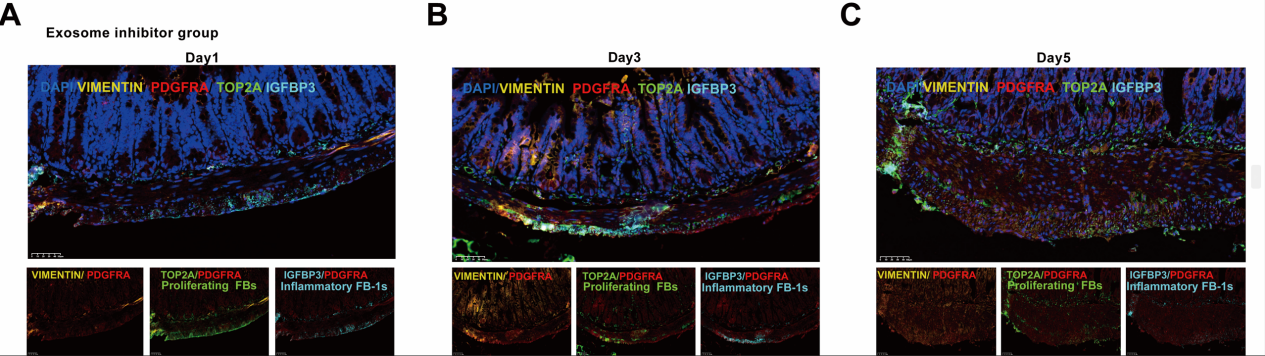


**Figure S14. Exosome depletion exhibits distinct effects on fibroblast subpopulations with subtype-specific responses.** (A-C) Immunofluorescence analysis of fibroblast phenotypes in the Exosome Depletion group on Days 1, 3, and 5 post-treatment. Images show proliferating FBs (TOP2A+/PDGFRA+) remained elevated while inflammatory FBs (IGFBP3+/PDGFRA+) were only partially suppressed.

**Table S1**

**Histopathological Evaluation (HE) Scoring Form**

| **HE Evaluation Grading System** | | |
| --- | --- | --- |
| **Grade (Number)** | **Category** | **Description** |
| 0 | Within Normal Range | Under research conditions, considering factors such as the animal's age, sex, and strain, the tissue is considered normal. Changes that appear under other conditions may be considered abnormal |
| 1 | Very Mild | Changes that just exceed the normal range |
| 2 | Mild | Lesions can be observed but are not yet severe |
| 3 | Moderate | Lesions are obvious and likely to become more severe |
| 4 | Severe | Lesions are very severe (affecting the entire tissue/organ) |
|  |  |  |

**Table S2**

**List of Antibodies Used in the Experiment**

| Table S2. List of antibodies | | | | |
| --- | --- | --- | --- | --- |
| **Primary antibodies for flow cytometry** | **Supplier** | **Catalogue number** | **Dilution** | **Experimental category** |
| FITC anti-mouse/human CD44 Antibody | BioLegend | 103022 | 1 μL per sample | for flow cytometry |
| BV421 anti-mouse/human CD11b | BioLegend | 101235 | 1 μL per sample | for flow cytometry |
| FITC anti-mouse CD45 Antibody | BioLegend | 103107 | 1 μL per sample | for flow cytometry |
| FITC anti-mouse/rat CD29 Antibody | BioLegend | 102205 | 1 μL per sample | for flow cytometry |
| Chromogranin A anti-rabbit antibody | Abcam | AB283265 | 1/50 | IF |
| Ki67 anti-mouse antibody | Abcam | AB279653 | 1/50 | IF |
| DAPI | Abcam | AB228549 | 1/100 | IF |
| Mucin 2 anti-mouse antibody | Santa Cruz | SC-7314 | 1/200 | IF |
| Villin anti-mouse Antibody | Santa Cruz | SC-58897 | 1/200 | IF |
| Chromogranin A anti-mouse antibody | Invitrogen | MA5-13096 | 1/200 | IF |
| Lysozyme Recombinant anti-Rabbit Monoclonal Antibody | Invitrogen | MA5-32154 | 1/100 | IF |
| Vimentin anti-rabbit antibody | Proteintech Group | 10366-1-AP | 1/400 | IF |
| PDGFRA anti-rabbit antibody | BOSTER Biological Technology Co., Ltd. | A00366 | 1/200 | IF |
| TOP2A anti-rabbit antibody | Proteintech Group | 24641-1-AP | 1/200 | IF |
| IGFBP3 anti-rabbit antibody | Proteintech Group | 10189-2-AP | 1/400 | IF |
| Col4a2anti-rabbit antibody | Proteintech Group | 55131-1-AP | 1/200 | IF |
| Col6a1anti-rabbit antibody | BOSTER Biological Technology Co., Ltd. | BM4748 | 1/100 | IF |
| Col6a2anti-rabbit antibody | Bioss Antibodies | bsm-61026R | 1/200 | IF |

| **Secondary Antibodies for Immunofluorescence (IF)** | **Supplier** | **Catalogue number** | **Dilution** |
| --- | --- | --- | --- |
| Goat pAb to rabbit lgG(Alexa Fluor 594) | Abcam | AB 150080 | 1/200 |
| Goat pAb to mouse lgG(Alexa Fluor 488) | Abcam | AB 150113 | 1/200 |
| Goat pAb to mouse lgG(Alexa Fluor 594) | Abcam | AB150116 | 1/200 |
